# Prevalence and determinants of inappropriate complementary feeding practices among children aged 6–23 months in Chiang Mai, Northern Thailand: a cross-sectional study

**DOI:** 10.1186/s12889-025-23293-z

**Published:** 2025-06-02

**Authors:** Krongporn Ongprasert, Jakarin Chawachat, Jukkrit Wungrath, Wuttipat Kiratipaisarl

**Affiliations:** 1 Department of Community Medicine, Faculty of Medicine, Chiang Mai University, Chiang Mai, Thailand; 2 Department of Computer Science, Faculty of Science, Chiang Mai University, Chiang Mai, Thailand; 3 Faculty of Public Health, Chiang Mai University, Chiang Mai, Chiang Mai, 50200 Thailand

**Keywords:** Complementary feeding, Caregiver support, Child nutrition, Dietary diversity

## Abstract

**Background:**

Inappropriate complementary feeding (CF) increases the risk of the triple burden of malnutrition, including undernutrition, micronutrient deficiencies, overweight and obesity. Chiang Mai, a major city in northern Thailand, is experiencing rapid urbanization and growing cultural diversity driven by tourism, which may influence CF practices. However, data on these practices remain limited, emphasizing the need for context-specific interventions.

**Methods:**

A cross-sectional study involving 1,122 caregivers of children aged 6–23 months was conducted between January and May 2024. Data were collected through face-to-face interviews and 24-hour dietary recalls. Multivariable log-binomial regression was used to identify factors associated with inappropriate CF practices.

**Results:**

Inappropriate CF was observed in 64.1% of the children. Dietary diversity increased with age, from an average of three food groups in infants aged 6–11 months to five food groups in children aged 18–23 months. Unhealthy dietary habits were more prevalent among children aged 18–23 months, with 38.4% consuming sweetened beverages and 47.8% consuming unhealthy foods. The key barriers included caregiver uncertainty about appropriate food textures (88.0%), food types (84.8%), and portion sizes (70.2%). The factors significantly associated with a greater risk of inappropriate CF included being a first-time caregiver (aRR: 1.18; 95% CI: 1.05–1.31) and the caregiver’s perception that the child preferred milk over solid foods (aRR: 1.32; 95% CI: 1.17–1.49). Additionally, receiving feeding information from family or friends (aRR: 1.15; 95% CI: 1.02–1.29) or from healthcare providers (aRR: 1.16; 95% CI: 1.02–1.33) was associated with a significantly greater risk than using unreliable online sources.

**Conclusion:**

Improving CF practices requires focused support for caregivers, particularly first-time parents. Interventions should promote dietary diversity, address common concerns related to CF, and ensure that caregivers have access to reliable nutritional information through accessible and trusted communication channels. Future research should evaluate the effectiveness of context-specific interventions tailored to local needs and caregiver characteristics.

**Supplementary Information:**

The online version contains supplementary material available at 10.1186/s12889-025-23293-z.

## Introduction

Complementary feeding (CF) refers to the introduction of solid or semisolid foods alongside breast milk or formula when these alone are no longer sufficient to meet an infant’s nutritional needs. This critical transitional period begins at six months of age and continues until 23 months [1]. Inappropriate CF remains a major public health issue in Southeast Asia (SEA), contributing to the region’s triple burden of malnutrition, which includes undernutrition, micronutrient deficiencies, childhood overweight and obesity [2]. Undernutrition, particularly stunting, affects approximately 25% of children under five years of age in SEA, whereas micronutrient deficiencies remain widespread, with an estimated 46% of children lacking adequate intake of essential nutrients such as iron, zinc, and vitamins A and D [2, 3]. Moreover, the prevalence of childhood overweight and obesity is increasing, especially among low-income populations, possibly due to increased access to inexpensive, energy-dense foods high in sugar and fat [4]. Currently, 7.5% of children under five in SEA are classified as overweight [2]. In Thailand, data from 2022 show a stunting prevalence of 8.7% and an overweight prevalence of 10.5% among children under five years of age [5].

In 2021, the World Health Organization (WHO) and the United Nations Children’s Fund (UNICEF) updated the indicators for assessing infant and young child feeding (IYCF) practices [6]. These key indicators include minimum dietary diversity (MDD), minimum meal frequency (MMF), and minimum acceptable diet (MAD). Globally, only 29% of children met the criteria for MDD, 53% for MMF, and 19% for MAD in 2021 [7]. In contrast, Thailand demonstrated markedly higher adherence to IYCF recommendations in 2022, with 76.7% of children meeting MDD, 81.2% meeting MMF, and 65.9% achieving MAD [5]. These figures indicate better nutritional status among Thai children than do global and regional averages. This progress may reflect sustained economic growth and the national child support grant [8]. Furthermore, national regulations such as the Control of Marketing Promotion of Infant and Young Child Food Act have supported optimal feeding practices by limiting the promotion of breast milk substitutes and other commercial baby food products [9].

Chiang Mai, located in northern Thailand, is characterized by its multiethnic population, cultural diversity, and significant rural-to-urban migration [10]. The city’s rapid urbanization and expanding tourism industry have contributed to a dynamic and evolving sociocultural environment [11]. Within this setting, caregivers often navigate a complex interplay between traditional beliefs shaped by longstanding cultural norms regarding child feeding and modern health and nutrition recommendations. As a result, CF practices in Chiang Mai are likely to be highly diverse and shaped by both historical influences and contemporary public health messaging [12]. However, data on these practices remain limited, emphasizing the need for context-specific interventions. This study aims to examine CF patterns among local caregivers. It also explores caregiver-reported barriers to appropriate feeding and identifies the main sources of information shaping their practices. Understanding these context-specific behaviors is essential for guiding effective policy design and implementing targeted programs to improve nutritional outcomes among infants and young children in this population.

## Methods

### Study setting

This study was conducted in Chiang Mai Province, Thailand, a major city in the northern region of the country. As of 2021, Thailand’s total population was 65,557,054, with Chiang Mai accounting for 1,628,164 residents [13]. In the same year, the national average monthly household income was 27,352 Thai Baht (808 USD), whereas Chiang Mai’s average was 22,963 Thai Baht (678 USD), ranking 43rd out of 77 provinces. Agriculture remains the primary source of employment in the province [14].

### Study design and period

A cross-sectional study was conducted between January and May 2024.

### Sample size and sampling procedure

The study targeted 15,629 children aged 6–23 months residing in Chiang Mai Province on the basis of 2019 demographic data [13]. The sample size was determined via an expected frequency of 55.5% on the basis of the proportion of children meeting the MAD indicator in previous studies in Thailand [15]. The calculation assumed a 97% confidence level, a 5% margin of error, and a design effect of 2.0, yielding a required sample size of 904 participants. To accommodate potential nonresponses or incomplete data, 20% oversampling was applied, increasing the final target sample size to at least 1,085 participants. Participants were recruited through convenience sampling at selected well-child clinics across three hospitals. These hospitals were purposively selected from the northern, central, and southern regions of Chiang Mai to represent the general population with access to basic medical care while intentionally excluding minority hill tribe populations.

### Data collection

Data were collected through structured one-on-one interviews via a questionnaire and a customized web-based application specifically developed for this study. The interviews were conducted by two trained interviewers with the supervision of the principal investigator. A structured questionnaire was designed for this study, covering five key domains: (1) caregiver and child sociodemographic characteristics and sources of CF information, (2) 24-hour dietary recall (open recall), (3) 24-hour dietary recall (list-based), (4) food and milk consumption frequency, and (5) barriers to CF. The definition of IYCF followed the 2021 WHO IYCF guidelines [6]. The dietary assessment was also adapted from the same source, with two local nutrition experts reviewing the original food list to ensure cultural relevance. Items not commonly consumed in Thailand, such as blackcurrant, nectarine, biltong, and beef jerky, were removed, while locally relevant foods such as sticky rice and instant noodles were added. A comprehensive list of the foods is included in the Supplementary File. The barriers to CF were incorporated based on findings from previous studies [2, 16–20]. A two-step 24-hour dietary recall method was used to assess dietary intake. In the first step, the caregivers provided an open recall of all the foods and beverages. When an item was reported, the interviewer entered a portion of the food name into the dropdown menu field in the web application for selection. In the second step, the interviewer read aloud a standardized food list for caregivers to verify consumption. Any unlisted foods were manually entered, after which a nutritionist reviewed and classified all manually recorded items.

### Operational definition

#### Inappropriate CF practices

In this study, inappropriate CF practices were defined as the failure to meet at least four of the six recommended healthy feeding indicators, which include continued breastfeeding (CBF), MDD, MMF, minimum milk feeding frequency (MMFF), MAD, and egg and/or flesh food consumption (EFF). In addition, children who were exposed to more than two of the following four unhealthy feeding practices were also classified under this definition. These practices include the consumption of sweetened beverages (SwB), unhealthy food consumption (UFC), zero vegetable or fruit consumption (ZVF), and bottle feeding (BoF).

### Food group consumption

Food group consumption was assessed on the basis of the child’s intake within the previous 24 h across eight predefined categories: (1) breast milk; (2) grains, roots, tubers, and plantains; (3) pulses, nuts, and seeds; (4) dairy products; (5) flesh foods; (6) eggs; (7) vitamin A-rich fruits and vegetables; and (8) other fruits and vegetables.

### Reliable source

Evidence-based information from peer-reviewed journals, official health organizations (e.g., WHO, CDC), or academic institutions.

### Unreliable source

Information lacking credibility, such as personal blogs, unverified social media posts, or biased websites.

### Data processing and analysis

The Kolmogorov‒Smirnov test for normality was carried out, and the child age and parent age were also corrected and reported as medians with quartiles 1 and 3. Moreover, the nonparametric Kruskal‒Wallis rank sum test was used instead of the parametric two-independent sample t test to derive the p value of these two determinants in the univariable analyses. Numerical data with a normal distribution are reported as the mean and standard deviation (SD). Data with a nonnormal distribution are reported as medians and interquartile ranges (IQRs). Categorical and ordinal data are presented as counts (n) and percentages (%). Inferential statistics, including two independent sample t tests, were used to compare two groups of normally distributed data. The rank-sum test was used to compare two groups of data, with at least one group demonstrating a nonnormal distribution. The Cochran‒Armitage nonparametric test for trends was used to examine trends in ordinal data. Multivariable log-binomial regression was used to identify the adjusted risk ratio (aRR) of factors associated with inappropriate CF practices. The set of determinants associated with these factors were based on the previously published literature regarding inappropriate CF in children and parents across previous literature [2, 16–20], including child age (3 categories): 6–11, 12–17, and 18–24 months; gender (binary): male or female; primary caregiver education (binary): lower than high school, or high school and above; primary caregiver marital status (binary): formal marriage or other; primary caregiver occupational status (4 categories): stay-at-home, full-time work with day shift, work with rotational shift, freelance work with flexible hours; number of previous children (binary): none or at least one child; household income (binary): 20,000 TH per month or > 20,000 TH per month; agree or strongly agree to each of the following major barriers to CF found in this population (four items, each with binary response): child preferred milk over food, lack of confidence in feeding type administered, lack of confidence in feeding amount administered, lack of confidence in feeding texture administered; sources of information (4 categories): face-to-face communication with family members or friends, face-to-face communication with healthcare providers, online unreliable sources, other (printed media of both nonacademic and academic). All the statistical analyses were carried out via the Stata version 18.0 statistical package (College Station, TX, USA). A *P* value less than 0.05 was considered statistically significant Table 1.

**Table 1:** Descriptive characteristics of the study population by appropriate complementary feeding practices

Characteristics	Total, *n* (%) (*N* = 1122)		CF practices, *n* (%)		*p* Value
			Inappropriate	Appropriate	
**CF practices**			719 (64.1)	403 (35.9)	
**Child**					
Age (months), median (Q1-Q3)		10 (7–17)	9 (7–15)	13 (9–19)	< 0.001**
6–11		607 (54.1)	436 (60.6)	171 (42.4)	< 0.001**
12–17		239 (21.3)	131 (18.2)	108 (26.8)	
18–24		276 (24.6)	152 (21.1)	124 (30.8)	
Gender					
Male		605 (53.9)	382 (53.1)	223 (55.3)	0.49
Female		517 (46.1)	337 (46.9)	180 (44.7)	
**Caregivers**					
Household guardian					
Both biological parents		456 (40.6)	318 (44.2)	138 (34.2)	0.004**
Mother only		365 (32.5)	210 (29.2)	155 (38.5)	
Maternal grandmother		169 (15.1)	106 (14.7)	63 (15.6)	
Others		132 (11.8)	85 (11.8)	47 (11.7)	
Primary caregiver age, median (Q1-Q3)		33 (28–43)	33 (27–42)	32 (28–45)	0.30
Primary caregiver education					
Lower than high school		665 (61.7%)	419 (60.1%)	246 (64.7%)	0.15
High school and above		412 (38.3%)	278 (39.9%)	134 (35.3%)	
Marital status (*n* = 1121)					
Formal Marriage		251 (22.4)	159 (22.1)	92 (22.9)	0.77
other		870 (77.6)	560 (77.9)	310 (77.1)	
Occupational status					
Stay-at-home		625 (55.7)	376 (52.3)	249 (61.8)	0.02*
Full-time work with day shift		239 (21.3)	162 (22.5)	77 (19.1)	
Work with rotational shift		58 (5.2)	40 (5.6)	18 (4.5)	
Freelance work with flexible hours		200 (17.8)	141 (19.6)	59 (14.6)	
Number of previous children					
None		170 (15.2)	138 (19.2)	32 (7.9)	< 0.001**
At least 1 child		951 (84.8)	580 (80.8)	371 (92.1)	
Household income (per month)					
≤ 20,000 TH (≤ 590 USD)^¥^		564 (50.7)	360 (50.6)	204 (50.9)	0.95
> 20,000 TH (> 590 USD) ^¥^		549 (49.3)	352 (49.4)	197 (49.1)	
**Barriers to CF** Agree/Strongly agree					
Confidence in feeding texture administered		987 (88.0%)	733 (87.5%)	254 (89.4%)	0.40
Confidence in feeding type administered		952 (84.8%)	707 (84.4%)	245 (86.3%)	0.50
Confidence in feeding amount administered		788 (70.2%)	592 (70.6%)	196 (69.0%)	0.60
**Child preferred milk over food**		**638 (56.9%)**	**546 (65.2%)**	**92 (32.4%)**	< 0.001**
Child denying new type of food		46 (4.1%)	35 (4.2%)	11 (3.9%)	> 0.99
Financial constraints		41 (3.7%)	28 (3.3%)	13 (4.6%)	0.36
Familial pressure		22 (2.0%)	15 (1.8%)	7 (2.5%)	0.46
**Sources of Information**					
Face-to-face communication family members or friends		276 (24.6%)	224 (26.8%)	52 (18.3%)	0.003**
Face-to-face communication with healthcare providers		419 (37.4%)	314 (37.6%)	105 (37.0%)	
Online unreliable sources		373 (33.3%)	267 (31.9%)	106 (37.3%)	
Other (printed media of both unreliable sources and academic)		52 (4.6%)	31 (3.7%)	21 (7.4%)	

Two-sample t-tests were used for normally distributed data. The Wilcoxon rank-sum test was applied when at least one group showed a nonnormal distribution. *Household Guardian: The individual residing with the child who holds primary responsibility for their care and supervision. Primary Caregiver: The person responsible for feeding the child. When both biological parents are listed as the household guardian*,* the mother is designated as the primary caregiver for consistency in record-keeping and analysis.* Exchange rate: ¥1.00 USD = 34.8 Thai Baht (TH) as of February 14, 2025. * *p* < 0.05; ** *p* < 0.01

## Results

### Participant characteristics

Table 1 presents the participant characteristics. Among the 1,122 participants, 64.1% of the children received inappropriate CF. The median age was significantly lower in the inappropriate group at 9 months (interquartile range [IQR] 7–15 months) than in the appropriate group at 13 months (IQR 9–19 months) (*p* < 0.001). A greater proportion of children aged 6–11 months was observed in the inappropriate group (60.6%) than in the appropriate group (42.4%) (*p* < 0.001). There was no significant difference in sex distribution between the groups (*p* = 0.49). Children living with both biological parents were more common in the inappropriate group (44.2%) than in the appropriate group (34.2%) (*p* = 0.004). No significant differences were observed in caregiver age (*p* = 0.30), educational attainment (*p* = 0.15), or marital status (*p* = 0.77). Stay-at-home caregivers were more common in the appropriate feeding group, accounting for 61.8%, than in the inappropriate feeding group, accounting for 52.3% (*p* = 0.02). Caregivers without previous children were more common in the inappropriate group, representing 19.2%, than in the appropriate group, with 7.9% (*p* < 0.001). Household income did not differ significantly between the groups (*p* = 0.95).

Among the reported barriers, the perception that the child preferred milk over food was significantly more common among caregivers in the inappropriate group (65.2%) than among those in the appropriate group (32.4%) (*p* < 0.001). Other common concerns, such as lack of confidence regarding feeding texture (88.0%), type (84.8%), and amount (70.2%), were frequently reported but showed no significant group differences. Less common barriers, including food refusal (4.1%), financial constraints (3.7%), and familial pressure (2.0%), were also not significantly different.

Receiving feeding advice through face-to-face communication with family members or friends was more common in the inappropriate group (26.8%) than in the appropriate group (18.3%) (*p* = 0.003). Other sources of information, including healthcare providers, online platforms, and printed materials, did not differ significantly between the two groups Table 2.

**Table 2:** Compliance with the WHO IYCF indicators and food group consumption across age groups

WHO IYCF indicators/food type	*n* (%), Response answer “yes”				*p* value for trend	
	All subjects (*n* = 1122)	6–11 months (*n* = 607)	12–17 months (*n* = 239)	18–24 months (*n* = 279)		
**WHO IYCF indicators**						
Continued breastfeeding (CBF)	514 (45.9)	369 (60.9)	85 (35.6)	60 (21.7)	< 0.001**	
Minimum dietary diversity (MDD)	305 (27.3)	73 (12.1)	112 (46.9)	120 (43.6)	< 0.001**	
Minimum meal frequency (MMF)	862 (76.8)	375 (61.8)	232 (97.1)	255 (92.4)	0.008**	
Minimum milk feeding frequency for nonbreastfed (MMFF)	575 (94.7)	232 (97.9)	152 (98.7)	191 (88.4%)	< 0.001**	
Minimum acceptable diet (MAD)	266 (23.8)	52 (8.6)	110 (46.0)	104 (37.8)	< 0.001**	
Egg and/or flesh food consumption (EFF)	779 (69.4)	326 (53.7)	212 (88.7)	241 (87.3)	0.045*	
Sweet beverage consumption (SwB)	161 (14.3)	18 (3.0)	37 (15.5)	106 (38.4)	< 0.001**	
Unhealthy food consumption (UFC)	289 (25.8)	51 (8.4)	106 (44.4)	132 (47.8)	0.021*	
Zero vegetable or fruit consumption (ZVF)	371 (33.1)	259 (42.7)	56 (23.4)	56 (20.3)	< 0.001**	
Bottle feeding (BoF)	633 (56.5)	378 (62.4)	159 (66.5)	96 (34.8)	0.078	
**Food Group Consumption**						
The average groups consumption, median (Q1-Q3)	4 (3–5)	3 (3–4)	5 (4–6)	5 (4–6)	< 0.001**	
Breast milk	514 (45.9)	369 (60.9)	85 (35.6)	60 (21.7)	< 0.001**	
Grains, roots, tubers	819 (73.0)	372 (61.3)	198 (82.8)	249 (90.2)	0.031*	
Pulses, nuts and seeds	22 (2.0%)	5 (0.8)	8 (3.3)	9 (3.3)	0.207	
Dairy products	470 (41.9)	107 (17.6)	161 (67.4)	202 (73.2)	< 0.001**	
Flesh foods	516 (46.0)	182 (30.0)	146 (61.1)	188 (68.1)	0.001**	
Eggs	558 (49.7)	210 (34.6)	179 (74.9)	169 (61.2)	< 0.001**	
Vitamin-A rich fruits and vegetables	549 (48.9)	280 (46.1)	139 (58.2)	130 (47.1)	0.002**	
Other fruits and vegetables.	425 (37.9)	155 (25.5)	109 (45.6)	161 (58.3)	0.308	

The Cochran–Armitage trend test was used to assess trends in ordinal data.* *p* < 0.05; ** *p* < 0.01

### Complementary feeding indicators and food group consumption

Data on the CF indicators and food group consumption data are presented in Table 2. Indicators with a prevalence exceeding 50% included MMF (76.8%), MMFF (94.7%), EFF (69.4%), and BoF (56.5%). Indicators with a prevalence below 50% included CBF (45.9%), MDD (27.3%), MAD (23.8%), SwB (14.3%), UFC (25.8%), and ZVF (33.1%). Significant age-related trends were observed across multiple indicators. The prevalence of CBF declined markedly from 60.9% among children aged 6–11 months to 21.7% at 18–24 months (*p* < 0.001). Similar downward trends were observed for the MMFF (from 97.9 to 88.4%, *p* < 0.001). and ZVF (from 42.7 to 20.3%, *p* < 0.001). In contrast, the prevalences of MDD (*p* < 0.001), MMF (*p* = 0.008), and MAD (*p* < 0.001) increased significantly with age. At 6–11 months, the MDD, MMF, and MAD rates were 12.1%, 61.8%, and 8.6%, respectively, increasing to 43.6%, 92.4% and 37.8%, respectively, at 18–24 months. EFF also increased with age, from 53.7 to 87.3% (*p* = 0.045).

Inappropriate feeding practices were more common among older children. The prevalence of SwB increased from 3.0 to 38.4% (*p* < 0.001), and that of UFC increased from 8.4 to 47.8% (*p* = 0.021). Although BoF tended to decrease with age, this difference was not statistically significant (*p* = 0.078).

Dietary variety, assessed by the number of food groups consumed, increased significantly with age (*p* < 0.001). The median number of food groups rose from 3 (IQR: 3–4) among children aged 6–11 months to 5 (IQR: 4–6) at 18–24 months. Among specific food groups, breast milk was the only one that significantly decreased with age, decreasing from 60.9% in the youngest group to 21.7% in the oldest group (*p* < 0.001). The most consumed food group across all ages was grains, roots, and tubers, with intake increasing from 61.3 to 90.2% (*p* = 0.031). The consumption of the four food groups increased significantly with age. These included dairy products (41.9%, *p* < 0.001), flesh foods (46.0%, *p* = 0.001), eggs (49.7%, *p* < 0.001) and vitamin A-rich fruits and vegetables (48.9%, *p* = 0.002). Other fruits and vegetables showed an increasing trend, although this difference was not statistically significant (*p* = 0.308). Pulses, nuts and seeds were the least consumed food group overall at 2.0% and showed no significant age-related differences (*p* = 0.207) Table 3.

**Table 3:** Multivariable log-binomial regression of factors associated with inappropriate complementary feeding practices among children aged 6–24 months (*N* = 1067)

Variables	Adjusted risk ratio (aRR) (95% CI)	*p* Value
**Child age (months)**		
6–11	1.14 (0.99, 1.31)	0.061
12–17	(reference)	
18–23	1.04 (0.89, 1.22)	0.612
**Child gender**		
male	(reference)	
Female	1.03 (0.94, 1.12)	0.507
**Parent educational level**		
Lower than high school	(reference)	
High school and above	1.02 (0.92, 1.14)	0.641
**Parent marital status**		
Not married	(reference)	
Married	1.07 (0.96, 1.20)	0.218
**Parent occupational status**		
Stay-at-home workers	(reference)	
Full-time work with day shift	1.08 (0.96, 1.21)	0.223
Work with rotational shift	1.04 (0.87, 1.25)	0.672
Freelance work with flexible hours	1.12 (1.00, 1.25)	0.056
**Number of previous children**		
**None**	1.18 (1.05, 1.31)	0.004**
At least one	(reference)	
**Family income per capita**		
≤ 20,000 TH (≤ 600 USD)	(reference)	
> 20,000 TH (> 600 USD)	1.02 (0.92, 1.12)	0.737
**Household guardian**		
Both biological parents	1.11 (0.98, 1.24)	0.090
Mother only	(reference)	
Maternal grandmother	1.05 (0.88, 1.24)	0.592
Others	1.07 (0.90, 1.26)	0.461
**Lack of confidence in feeding type administered**		
Not agree or neutral	(reference)	
Agree to strongly agree	0.89 (0.78, 1.02)	0.085
**Lack of confidence in feeding amount administered**		
Not agree or neutral	(reference)	
Agree to strongly agree	1.06 (0.95, 1.18)	0.283
**Lack of confidence in feeding texture administered**		
Not agree or neutral	(reference)	
Agree to strongly agree	0.95 (0.83, 1.08)	0.417
**Child preferred milk over food**		
Not agree or neutral	(reference)	
Agree to strongly agree	1.32 (1.17, 1.49)	< 0.001**
**Sources of Information used to obtain information on CF**		
Face-to-face communication with family members or friends	1.15 (1.02, 1.29)	0.024*
Face-to-face communication with healthcare providers	1.16 (1.02, 1.33)	0.025*
Online unreliable sources.	(reference)	
Other (printed media of both non unreliable and reliable sources)	1.04 (0.85, 1.28)	0.688

Multivariable log-binomial regression was used to estimate adjusted risk ratios (aRRs) for factors associated with inappropriate complementary feeding. Models were adjusted for child age and gender, parental education, marital and employment status, number of previous children, household income, guardian type, key feeding barriers, and sources of information

Exchange rate: ¥1.00 USD = 34.8 Thai Baht (TH) as of February 14, 2025. * *p* < 0.05; ** *p* < 0.01

### Factors associated with inappropriate complementary feeding practice

Multivariable log-binomial regression was performed to identify factors associated with inappropriate complementary feeding (CF) among 1,067 children aged 6–24 months, as presented in Table 3. Caregivers with no previous child-rearing experience had a greater risk of inappropriate feeding than did those with at least one child (aRR = 1.18; 95% CI: 1.05–1.31; *p* = 0.004). Caregivers who reported that their child preferred milk over food were more likely to engage in inappropriate feeding practices (aRR = 1.32; 95% CI: 1.17–1.49; *p* < 0.001). Receiving feeding information through face-to-face communication with family or friends (aRR = 1.15; 95% CI: 1.02–1.29; *p* = 0.024) and from healthcare providers (aRR = 1.16; 95% CI: 1.02–1.33; *p* = 0.025) was associated with a greater risk of inappropriate feeding than relying on online sources was considered unreliable. No significant associations were detected for child age, sex, parental education, marital status, occupational status, household guardian arrangement, household income, or caregiver confidence in feeding practices.

## Discussion

This study examined CF practices among children aged 6 to 24 months in Chiang Mai, Thailand, based on 24-hour dietary recall. Approximately two-thirds of the children were classified as having inappropriate CF. Among the three key CF indicators, the prevalences of MDD, MMF, and MAD were 27.3%, 76.8%, and 23.8%, respectively. These rates were substantially lower than those reported in neighboring upper-middle-income countries and national data from Thailand [21]. Between 2022 and 2024, the MDD prevalence rates were 81.9% in Malaysia, 54.3% in Indonesia, and 76.7% in Thailand. The MMF ranged from 63.4 to 81.2%, and the MAD ranged from 37.6 to 60.8% [5, 19, 22]. Although Thailand has adopted national and international guidelines that promote dietary diversity from an early age [6, 23–25], the prevalence of MDD remains suboptimal in this study. One possible explanation is the lower average household income among study participants than among national participants [14], which has been associated with a greater risk of inappropriate CF practices [16, 26]. Additionally, persistent traditional beliefs that are inconsistent with current feeding guidelines may influence caregiver behaviors and hinder adherence to recommended practices [27].

Among children aged 6 to 11 months, approximately two-thirds were breastfed. While this prevalence is higher than that of older age groups, it remains below the recommended level. During this critical stage of growth and development, breast milk is expected to provide at least half of a child’s energy needs [28]. This indicates the need for targeted interventions to promote continued breastfeeding throughout the first year of life. In children aged 12 to 24 months, a decline in breast milk consumption was observed. However, this was not matched by an improvement in overall dietary diversity, despite this being a critical period of rapid growth when a variety of solid foods should serve as the primary source of energy and nutrients [6]. The study revealed persistent gaps in the consumption of specific food groups. For example, only around two-thirds of children in this age group consumed flesh foods and egg. Previous studies in the Chiang Mai population have identified common caregiver misconceptions, including the belief that animal-source proteins are unsuitable for infants [27]. Addressing these misconceptions through culturally sensitive education and community engagement may be essential to improving feeding practices and supporting optimal growth and development. Pulses, nuts, and seeds represented the least consumed food group, reported in only 2% of the children. Similarly, low consumption was reported in Vietnam (6.8%) and the Philippines (5.6%) (11, 26). Efforts to promote this food group should include caregiver education on nutritional benefits and safe preparation, such as pureeing or mashing into a paste to reduce choking risk. The demonstration of local recipes has also been shown to improve feeding practices [29, 30].

Unhealthy feeding practices, such as the consumption of SwB and UFC, became more prevalent as children grew older. By 18 to 24 months of age, one-third of the children consumed sweetened beverages, and nearly half consumed unhealthy foods. These patterns are consistent with broader trends in SEA, where the consumption of processed foods is increasing due to aggressive marketing, low prices, and the widespread availability of snacks high in sugar, salt, and fat [2]. Health promotion efforts should focus not only on supporting the timely introduction of healthy foods but also on minimizing children’s exposure to unhealthy dietary options.

Inappropriate CF practices were significantly associated with specific caregiver characteristics. First-time caregivers were particularly vulnerable, as supported by previous studies [16, 18]. These caregivers may have lower confidence, less parenting experience, and a limited understanding of feeding guidelines [31, 32]. Another significant factor was the caregiver’s perception that the child preferred milk over food. This belief may stem from a limited understanding of normal feeding behaviors, such as gagging or early resistance to textured foods, which typically decrease by 12 months of age [24, 33]. Moreover, this misconception may be reinforced by persuasive promotional messaging that emphasizes the benefits of milk while failing to offer clear guidance on appropriate quantities or the timing of solid food introduction. A previous study reported that 50% of preschool-aged children in Thailand consumed more than 500 milliliters of milk per day, influenced by the belief that milk is the most essential source of nutrition [34]. Although not statistically significant in our analysis, many caregivers reported uncertainty about food textures, portion sizes, and appropriate food types. This concern is consistent with findings from a previous study, which reported that 69% of caregivers expressed a need for portion-size examples, 59% requested guidance on suitable foods, and 52% sought advice on food preparation [35]. Our findings emphasize the importance of strengthening caregiver education through the provision of clear and practical information.

The results showed that caregivers who received information through face-to-face communication with family members, friends, or healthcare providers were more likely to report inappropriate CF practices than those who relied on the internet or social media sources, even when the latter were considered unreliable. This trend may be attributed to the reliance on advice from relatives, which is often based on personal experience and does not always align with current dietary guidelines. It also suggests a lack of sufficient opportunities for in-depth counseling during routine child health visits in Thailand. Previous studies suggest that caregivers often leave these visits with unresolved questions, leading them to independently seek additional information from other sources [35]. Online platforms have become an increasingly popular source of information among caregivers. Research conducted in Thailand has shown that caregivers who actively engage with social media are more likely to provide fruits and vegetables to their children [36]. However, the quality of online content varies widely, and some digital information may conflict with established nutritional guidelines [37, 38]. Our study did not find a significant association between child age and inappropriate CF practices, in contrast to previous studies [16, 18, 20]. This difference may be explained by our inclusion of SwB and UFC as indicators of inappropriate feeding, which are less prevalent among younger children.

### Limitations

The definition of inappropriate CF used in this study was developed internally to capture both desirable and undesirable feeding behaviors. An overlap exists between specific indicators, as the MAD is derived from MDD and MMF. Including all three indicators separately may have led to repeated counting, which could result in an overestimation of the prevalence of inappropriate feeding. Additionally, the scoring system assigned equal weight to both positive and negative feeding behaviors, even though there were more items classified as healthy (six items) than unhealthy (four items). This approach could have led to misclassification, particularly in borderline cases. For example, a child with a high score in healthy practices but also exhibiting multiple unhealthy behaviors might still be classified as having inappropriate CF. Future research should consider refining the scoring model, possibly by applying a weighted or tiered system to better reflect the relative impact of different feeding behaviors on child health outcomes. Moreover, the participants were purposively selected from three hospitals that provide basic medical services, reflecting a population with access to standard healthcare. However, this sampling strategy may limit the generalizability of the results. Therefore, the findings should be interpreted with caution.

## Conclusion

This study reports a high prevalence of inappropriate CF practices among children aged 6–24 months in Chiang Mai, Thailand. However, the frequency of meals was generally low, particularly among younger children. The consumption of pulses, nuts, and seeds was especially limited. Key risk factors included first-time caregiving, caregivers’ perception that the child preferred milk over solid foods, and reliance on face-to-face information from healthcare providers or family and friends rather than online sources. To improve CF practices, targeted interventions should support caregivers, particularly those with limited experience. Efforts should focus on promoting early dietary diversity. Moreover, delivering evidence-based nutritional guidance through accessible and trusted communication channels is essential to support informed caregiver decisions and encourage appropriate feeding behaviors.

## Electronic supplementary material

Below is the link to the electronic supplementary material.

Supplementary Material 1

## Data Availability

The research data supporting the results of this manuscript are available upon request. Interested parties can contact the corresponding author directly for access to the data.

## Abbreviations

CF: Complementary feeding
SEA: Southeast Asia
WHO: World Health Organization
UNICEF: United Nations Children’s Fund
IYCF: Infant and young child feeding practices
MDD: Minimum Dietary Diversity
MMF: Minimum meal frequency
MAD: Minimum Acceptable Diet
CBF: Continued breastfeeding
MMFF: Minimum Milk Feeding Frequency
EFF: Egg and/or Flesh Food Consumption
SwB: Sweet beverage consumption
UFC: Unhealthy Food Consumption
ZVF: Zero Vegetable or Fruit Consumption
BoF: Bottle feeding
SD: Standard deviation
IQR: Interquartile range
aRR: Adjusted risk ratio
TH: Thai Baht
