# Supplementary material for: Prevalence and determinants of inappropriate complementary feeding practices among children aged 6–23 months in Chiang Mai, Northern Thailand: a cross-sectional study
Source: BMC Public Health. 2025 Jun 2;25:2043. doi: 10.1186/s12889-025-23293-z (PMC12128354; doi:10.1186/s12889-025-23293-z)
Supplement: Supplementary file 1 — Supplementary Material 1 [file 12889_2025_23293_MOESM1_ESM.docx]

**Supplementary Document 1**
*(Dataset for Developing the Web-based Application)*

**Home Page**

| [alert box : inform participant]  Explain that the questionnaire includes the following three objectives:   1. To collect data on your child's consumption of nutrient-rich foods (e.g., milk, grains, meat food, eggs, and fruits and vegetables) and unhealthy meals and drinks (e.g., sweet beverages, sentinel sweet foods, sentinel fried and salty foods) 2. To collect data regarding the frequency of your child's meals, milk feeds, and snacks. 3. To collect data about mothers' (or primary caregivers') primary sources of information about feeding practices and the influence of influential individuals on the feeding practice. |
| --- |
| [alert box : inform participant]  Explain that the questionnaire includes the following four components:  1. The demographics and characteristics of the mothers, child and / or primary caregiver.  2. The nutrient-rich and unhealthy foods consumed within the past twenty-four hours by your child. I will begin by asking you to recall the foods your child ate between waking up and breakfast yesterday. After that, I will read the names of the foods to help you recall everything your child ate.  3. The frequency of your child's meals, milk feeds, and snacks within the previous twenty-four hours.  4. Barriers to complementary feeding are imposed by the maternal perspective |

**PART 1. Characteristic (A)**

|  | **A** | **Question** | **Response** |
| --- | --- | --- | --- |
| **Primary Caregiver** |  |  |  |
| Relationship of the respondent to the child | A1 | A1 What is your relationship with the child?  A. Mother  B. Father  C. Grandparent  D. Aunt /Uncle  E. Other | [A] or [B] or [C] or [D] or [E] |
| [alert box : inform participant] |  | Please note that the following question refers to the mother of this child. |  |
| Age | A2 | A2 How old is the primary caregiver?  A. Year  B. Don’t know | [A] Number (age)  I___I  [B] code = Z |
| 18-25 |  |  |  |
| 26-35 |  |  |  |
| Over 35 |  |  |  |
| Education level | A3 | A3 What is the highest degree or level of education the primary caregiver has completed?  A. 6th grade or less  B. 6th -12th grade  C. Some college, no degree  D. Bachelor's Degree or higher  E. Don’t know | [A] or [B] or [C] or [D] or [E] |
|  |  |  |  |
| Marital status | A4 | A4 Is the primary caregiver's marital status defined as married?  1. Yes  0. No  9. Don’t know | [1] or [0] or [9] |
| Single |  |  |  |
| Married |  |  |  |
| Occupational status (shiftwork type) of the mother | A5 | A5 What is the employment status of the primary caregiver?  A. Stay-at-home mothers  B. Full-time work with day shift  C. Work with rotational shift  D. Freelance work with flexible hours  E. Other  F. Don’t know | [A] or [B] or [C] or [D] or [E] or [F] |
| Stay-at-home mothers |  |  |  |
| Full-time work with day shift |  |  |  |
| Work with rotational shift |  |  |  |
| Freelance work with flexible hours |  |  |  |
| Other |  |  |  |
| Number of previous children | A6 | A6 How many children has the primary caregiver had previously? (not include this child)  A. Number  B. Don’t known | [A] Number (of previously child)  I___I  [B] |
| None |  |  |  |
| 1 |  |  |  |
| 2, 3 |  |  |  |
| Over 3 |  |  |  |
| The most influential sources used to obtain information on caregiver complementary feeding practices. | A7 | A7 What is the most influential sources used to obtain information on primary caregiver complementary feeding practices? | [A] or [B] or [C] or [D] or [E] or [F] or [G] ] |
| face-to-face communication with healthcare providers |  | A.face-to-face communication with healthcare providers  B.face-to-face communication family members or friends  C.Internet or social media of academic Institutions or professional institutions  E.Internet or social media of non-academic sources (e.g. Online Communities ,personal Facebook page, Parenting Magazines)  F.Printed media of academic Institutions or professional institutions  G.Printed media of non-academic sources |  |
| face-to-face communication family members or friends |  |  |  |
| Internet or social media of academic Institutions or professional institutions |  |  |  |
| Internet or social media of non-academic sources (e.g. Online Communities ,personal Facebook page, Parenting Magazines) |  |  |  |
| Printed media of academic Institutions or professional institutions |  |  |  |
| Printed media of non-academic sources |  |  |  |
| **Children** |  |  |  |
| Gender | A8 | A8 What is the gender of your child?  A. Male  B. Female | [A] or [B] |
| Male |  |  |  |
| Female |  |  |  |
| Age of child (months) | A9.1  A9.2  A9.3 | A9 What is the date of your child's birth?  (day, month, and year) | Birthday  Day I_ _I  Month I_ _I  Year I _ _ _ _I |
| 12-18 |  |  |  |
| 18-24 |  |  |  |
| **Household** |  |  |  |
| **Family income per capita** | A11 | A11 What is average monthly household income? | Number (baths)  I____I |
|  |  |  |  |
| **Main caregiver to the child** | A12 | A12 Who is the child's main caregiver?  A. Both biological parents  B. Mother only  C. Maternal grandmother  D. Other | [A] or [B] or [C] or [D] or [E] |
| Both biological parents |  |  |  |
| Mother only |  |  |  |
| Maternal grandmother |  |  |  |
| Other |  |  |  |

PART 2. Food: OPEN RECALL (B)

|  | **Question** |
| --- | --- |
| B | B. Open recall |
|  | [alert box : inform participant]  Now I would like to ask you about everything that [NAME] ate yesterday during the day or at night.  This recall period starts from when the [NAME] awoke the previous day and extends throughout the day and night until [NAME] waking today, for a total time period of approximately 24 hours.  I am interested in foods your child ate whether at home or somewhere else. I will ask you about different types of foods, and I would like to know whether your child ate the food even if it was combined with other foods in a mixed dish *[like fried rice, noodles, or porridge]* will be asking what the child drank and ate, but not how much. |
|  | [alert box : Note for interviewer]  1. Repeat this set of questions and break them up into periods to get participants to think about what they feed their children.  2. If a mixed dish is mentioned, the interviewer should probe, "What were the main ingredients in MIXED DISH which [NAME] ate?" and record only those ingredients that the child really ate.  3. Consumption of any amount of food or beverage from a food group is sufficient to “count”, i.e., there is no minimum quantity. |
|  | [alert box : inform participant]  The open/ recall question.  - Please think about the milk, beverage, or food that [NAME] ate between waking up and eating breakfast yesterday.  - Did [NAME] eat anything at that time?  - Anything else? |
|  | List of time periods  B1. The child woke up previous morning, before breakfast  B2.Breakfast  B3.Before lunch  B4.Lunch  B5.Before dinner  B6.Dinner  B7.After dinner  B8.Night feed |

PART 3 Food: LIST BASE (C-E)

| **Indicators / short name** |  | **Binomial name OR genus** | **Family** | **Food list** | **Response** |
| --- | --- | --- | --- | --- | --- |
|  | **C** |  |  | [alert box : inform participant]  Just to make sure, did [NAME] eat [FOOD GROUP ITEMS] yesterday during the day or the night? |  |
| 1. Continued breastfeeding / CBF | **C1** |  |  | **(C1) Breast milk** |  |
|  |  |  |  | C1 Breast milk  1. yes  0. no | [0] or [1] |
| 2. Minimum dietary diversity / MDD | **C2** |  |  | **(C2) Grains, roots, tubers** |  |
|  |  |  |  | [alert box : Note for interviewer]  Sweet biscuits, cakes, pies, sweet donuts and other grain-based confectionery are not included and are classified with “Sentinel sweet foods”. Savoury doughnuts, instant noodles, and certain other fried and salty grain-based foods are classified with “Sentinel fried and salty foods” (…) |  |
|  |  |  |  | **(C2/set 1) C.2.1 Unprocessed food : Grains** |  |
|  |  | Triticum | Poaceae | C2.1.1 Rice | [0] or [1] |
|  |  | Oryza sativa | Poaceae | C2.1.2 Wheat | [0] or [1] |
|  |  | Zea mays | Poaceae | C2.1.3 Corn | [0] or [1] |
|  |  | Avena sativa | Poaceae | C2.1.4 Oats | [0] or [1] |
|  |  | Chenopodium quinoa | Amaranthaceae | C2.1.5 Quinoa | [0] or [1] |
|  |  |  |  | **(C2/set 2) C.2.2 Unprocessed food : Roots, Tubers** |  |
|  |  | Solanum tubeosum | Cactaceae | C2.2.1 Potatoes | [0] or [1] |
|  |  | Manihot esculentum | Moraceae | C2.2.2 Cassava | [0] or [1] |
|  |  | Ipomoea batatas | Apicaceae | C2.2.3 Sweet potato | [0] or [1] |
|  |  | Colocasia esculenta | Solanaceae | C2.2.4 Taro root | [0] or [1] |
|  |  | Eleocharis dulcis | Araceae | C2.2.5 Water chestnut | [0] or [1] |
|  |  | Nelumbo nucifera | Asteraceae | C2.2.6 Lotus root | [0] or [1] |
|  |  | Pachyorhizuserosus | Euphorbiaceae | C2.2.7 Jicama/Yambean | [0] or [1] |
|  |  | Amorhophallus paeoniifoleus | Cannaceae | C2.2.8 Elephant foot yam | [0] or [1] |
|  |  |  |  | **(C2/set 3) C2.3 Rice dishes*** |  |
|  |  |  |  | C2.3.1 Steamed rice***** | [0] or [1] |
|  |  |  |  | C2.3.2 Stick rice* | [0] or [1] |
|  |  |  |  | C2.3.3 Porridge* | [0] or [1] |
|  |  |  |  | C2.3.4 Boiled rice* | [0] or [1] |
|  |  |  |  | C2.3.5 Fried rice* | [0] or [1] |
|  |  |  |  | C2.3.6 Coconut stick rice* | [0] or [1] |
|  |  |  |  | C2.3.7 Steamed sticky rice cake with banana* | [0] or [1] |
|  |  |  |  | C2.3.8 Rice ball* | [0] or [1] |
|  |  |  |  | **(C2/set 4) C2.4 Product contains flour sourced from grains** |  |
|  |  |  |  | C2.4.1 Breads | [0] or [1] |
|  |  |  |  | C2.4.2 spaghetti | [0] or [1] |
|  |  |  |  | C2.4.3 Noodle | [0] or [1] |
|  |  |  |  | C2.4.4 Soba* | [0] or [1] |
|  |  |  |  | C2.4.5 pasta | [0] or [1] |
|  |  |  |  | **(C2/set 5) C.2.5 Product of Grains, roots, tubers** |  |
|  |  |  |  | C2.5.1 Peanut butter | [0] or [1] |
|  |  |  |  | C2.5.2 Tofu | [0] or [1] |
|  | **C3** |  |  | **C3 Pulses Nuts and Seeds** |  |
|  |  |  |  | [alert box : Note for interviewer]  This group includes: Mature seeds of beans/peas and all dried pulses are classified in the group “Beans, peas, lentils, nuts or seeds”.  This group “not” includes  legumes when the immature, fresh or green pod is consumed (e.g., fresh peas, snow peas, snap peas or green beans), which are classified in the group “other vegetable” |  |
|  |  |  |  | **(C3/set 1) C3.1 Pulses (beans, peas)** |  |
|  |  | Pisum sativum | Fabaceae | C3.1.1 Pea | [0] or [1] |
|  |  | Glycine max | Fabaceae | C3.1.2 Soybean | [0] or [1] |
|  |  | Vicia faba | Fabaceae | C3.1.3 Broad bean | [0] or [1] |
|  |  | Cicer arietinum | Fabaceae | C3.1.4 Chickpeas | [0] or [1] |
|  |  | Phaseolus vulgaris | Fabaceae | C3.1.5 Black bean | [0] or [1] |
|  |  | Phaseolus vulgaris | Fabaceae | C3.1.6 Kidney bean | [0] or [1] |
|  |  | Cajanus | Fabaceae | C3.1.7 Pigeon pea | [0] or [1] |
|  |  | Lablab purpureus | Fabaceae | C3.1.8 Hyacinth bean | [0] or [1] |
|  |  | Vigna radiata | Fabaceae | C3.1.9 Mung bean | [0] or [1] |
|  |  |  |  | **(C3/set 2) C3.2** **Nuts** |  |
|  |  | Arachis hypogaea | fabaceae | C3.2.1 Peanut/groundnut | [0] or [1] |
|  |  | Prunus dulcis | Rosaceae | C3.2.2 Almond | [0] or [1] |
|  |  | Anacardium occidentale | Anacardiaceae | C3.2.3 Cashew nut | [0] or [1] |
|  |  | Castanea | Fegaceae | C3.2.4 Chestnut | [0] or [1] |
|  |  |  |  | **(C3/set 3) C3.3**  **Seed** |  |
|  |  | Helianthus | Asteraceae | C3.3.1 Sunflower seed | [0] or [1] |
|  |  | Sesamum indicum | Pedaliacae | C3.3.2 Sesame seed | [0] or [1] |
|  |  | Cucurbita | Cucurbitaceae | C3.3.3 Pumpkin seed | [0] or [1] |
|  |  | Citrullus lanatus | Cucurbitaceae | C3.3.4 Melon seeds | [0] or [1] |
|  |  | Linum usitatissimum | Linaceae | C3.3.5 Flaxseed | [0] or [1] |
|  |  | Salvia hispanica | Limiaceae | C3.3.6 Chia seed | [0] or [1] |
|  | **C4** |  |  | **C4 Dairy products (milk, infant formula, yogurt, cheese)** |  |
|  |  |  |  | C4.1 Cow milk | [0] or [1] |
|  |  |  |  | C4.2 Goat milk | [0] or [1] |
|  |  |  |  | C4.3 Formula milk | [0] or [1] |
|  |  |  |  | C4.4 Yogurt | [0] or [1] |
|  |  |  |  | C4.5 Cheeses | [0] or [1] |
|  | **C5** |  |  | **C5 Flesh foods (meat, fish, poultry, organ meats)** |  |
|  |  |  |  | [alert box : Note for interviewer]  - Flesh meats prepared using any cooking method are included in this group; that is, even if deep-fried, meat is included here and is not included with “unhealthy” fried and salty foods, because of the importance for IYC of consuming nutrient-dense flesh foods with bioavailable micronutrients.  - Processed meat is grouped separately from unprocessed meat for two reasons. (Processed meat is defined as transformed through salting, curing, fermentation, smoking or other processes to enhance flavour or improve preservation) e.g., Hot dogs, Ham, Sausages, Biltong or beef jerky, Canned meat |  |
|  |  |  |  | **(C5/set 1) C5.1 Meat and poultry** |  |
|  |  |  |  | C5.1.1 Pork | [0] or [1] |
|  |  |  |  | C5.1.2 Beef | [0] or [1] |
|  |  |  |  | C5.1.3 Chicken | [0] or [1] |
|  |  |  |  | C5.1.4 Duck | [0] or [1] |
|  |  |  |  | **(C5/set 2) C5.2 Fish and shellfish** |  |
|  |  |  |  | [alert box : Note for interviewer]  This group includes fish, shellfish and seafood from both marine and freshwater environments. |  |
|  |  |  |  | C5.2.1 Shrimp, lobster, crayfish | [0] or [1] |
|  |  |  |  | C5.2.2 Clams, mussels, oysters and scallops | [0] or [1] |
|  |  |  |  | C5.2.3 Crabs | [0] or [1] |
|  |  |  |  | C5.2.4 Fish | [0] or [1] |
|  |  |  |  | C5.2.5 Octopus, squid and cuttlefish | [0] or [1] |
|  |  |  |  | **3. Organ meats** |  |
|  |  |  |  | C5.3.1 Blood sausage, other blood products | [0] or [1] |
|  |  |  |  | C5.3.2 Liver | [0] or [1] |
|  |  |  |  | C5.3.3 Gizzard | [0] or [1] |
|  | **C6** |  |  | **C6 Egg** |  |
|  |  |  |  | [alert box : Note for interviewer]  All kinds of eggs. Include even if only the white or only the yolk is eaten. |  |
|  |  |  |  | C6 egg | [0] or [1] |
|  | **C7** |  |  | **C7 Vitamin-A rich fruits and vegetables** |  |
|  |  |  |  | **(C7/set 1) C7.1 Dark green vitamin A-rich leafy vegetables** |  |
|  |  | Ipoemoea aquatica | Convolvilaceae | C7.1.1 Water spinach | [0] or [1] |
|  |  | Brassica oleracea | Brassicaceae | C7.1.2 Broccoli | [0] or [1] |
|  |  | Coccinia grandis | Cucurbitacea | C7.1.3 Ivy gourd | [0] or [1] |
|  |  | Brassica rapa | Brassicaceae | C7.1.3 Broccoli rabe | [0] or [1] |
|  |  | Brassica rapa | Brassicaceae | C7.1.4 Amaranth greens | [0] or [1] |
|  |  | Spinous oleeacea | Amaranthaceae | C7.1.5 bok choy | [0] or [1] |
|  |  | Sinapsis alba | Brassicaceae | C7.1.6 Spinach | [0] or [1] |
|  |  | Lactuca sativa | Asteraceae | C7.1.7 Mustard greens | [0] or [1] |
|  |  | Abelmoschus esculentus | Malvaceae | C7.1.8 Lettuce | [0] or [1] |
|  |  | Eruca sativa | Brassicaceae | C7.1.9 Okra greens | [0] or [1] |
|  |  | Brassica oleracea | Brassicaceae | C7.1.10 Arugula | [0] or [1] |
|  |  |  |  | **(C7/set 2) C7.2 Vitamin A-rich yellow/orange-fleshed fruits** |  |
|  |  | Mangifera indica | Anacardiaceae | C7.2.1 Mango, ripe *(fresh and dried)* | [0] or [1] |
|  |  | Carica papaya | Caricaceae | C7.2.2 Papaya, ripe *(fresh and dried)* | [0] or [1] |
|  |  | Cucumis melo | Cucurbitaceae | C7.2.3 Cantaloupe melon *(ripe)* | [0] or [1] |
|  |  | Cucumis melo | Cucurbitaceae | C7.2.4 Musk melon | [0] or [1] |
|  |  | Passiflora edulis | Passifloracceae | C7.2.5 Passion fruit *(ripe)* | [0] or [1] |
|  |  | Spondias mombin | Anacardiaceae | C7.2.6 Hog plum | [0] or [1] |
|  |  | Disospyros kaki | Ebenaceae | C7.2.7 Persimmon *(ripe)* | [0] or [1] |
|  | **C8** |  |  | **C8 Other fruits and vegetable** |  |
|  |  |  |  | **(C8/set 1) C8.1 Other vegetable** |  |
|  |  |  |  | [alert box : Note for interviewer]  This group includes  [1] This group includes all vegetables other than those classified as vitamin A-rich.  [2] legumes when the immature, fresh or green pod is consumed (e.g., fresh peas, snow peas, snap peas or green beans).  [3] It includes stems, fruits and flowers of plants when generally consumed in savoury dishes and considered as vegetables in culinary systems. So, for example, avocado, cucumber, tomato and okra (all fruits in botanical terms) are included as “Other vegetables”.  [4] Fresh, frozen and canned vegetables |  |
|  |  |  |  | [alert box : Note for interviewer]  [1] Foods made with mature beans or peas (seed only) and it does not include dried beans, peas and lentils, which are classified in the group “Beans, peas, lentils, nuts or seeds”.  [2] high carbohydrate “starchy” roots and tubers such as white potatoes, white yams, cassava and cocoyam, which are classified in the “White/pale starchy roots, tubers and plantains” group. |  |
|  |  | Solanum lypopersicum | Solanaceae | C8.1.1 Tomato | [0] or [1] |
|  |  | Cucurbita Species | Cucurbitaceae | C8.1.2 Cucumbers | [0] or [1] |
|  |  | Brassica oleracea | Brassicaceae | C8.1.3 Cabbage | [0] or [1] |
|  |  | Brassica oleracea | Brassicaceae | C8.1.4 Cauliflower | [0] or [1] |
|  |  | Brassica oleracea | Brassicaceae | C8.1.5 Kohlrabi | [0] or [1] |
|  |  | Zea mays | Poaceae | C8.1.6 Corn (fresh) | [0] or [1] |
|  |  | Benincasa hispida | Cucurbitaceae | C8.1.7 Winter melon | [0] or [1] |
|  |  | Solanum melongena | Solanaceae | C8.1.8 Eggplan | [0] or [1] |
|  |  | Persea americana | Lauraceae | C8.1.9 Avocado | [0] or [1] |
|  |  | Apium graveolens | Apiaceae | C8.1.10 Celery | [0] or [1] |
|  |  | Sechium | Cucurbitaceae | C8.1.11 Chayote | [0] or [1] |
|  |  | Luffa acutanguka | Cucurbitaceae | C8.1.12 Luffa | [0] or [1] |
|  |  | Trichosanthes cucumerina | Cucurbiticeae | C8.1.13 Snake gourd | [0] or [1] |
|  |  | Agaricus bisporus | Agaricaceae | C8.1.14 Mushroom | [0] or [1] |
|  |  | Phaseolus, others | Fabaceae | C8.1.15 Beans and Peas (various) when eaten as fresh pods | [0] or [1] |
|  |  | Bambusavulgaris | Poaceae | C8.1.16 Bamboo shoots | [0] or [1] |
|  |  | Luctuca sativa | Asteraceae | C8.1.17 Lettuce | [0] or [1] |
|  |  | Allium ampeloprasum | Alliaceae | C8.1.18 Leek | [0] or [1] |
|  |  | Cucurbita pepo | Cucurbitaceae | C8.1.19 Zucchini | [0] or [1] |
|  |  | Raphanus sativus | Brassicaceae | C8.1.20 Radish | [0] or [1] |
|  |  | Abelmoschus esculentus | Malvaceae | C8.1.21 Okra | [0] or [1] |
|  |  | Brassica oleracea | Brassicaceae | C8.1.22 Brussels sprouts | [0] or [1] |
|  |  | Beta vulgaris | Chenopodiaceae | C8.1.23 Beet | [0] or [1] |
|  |  | Asparagus officinales | Asparagaceae | C8.1.24 Asparagus | [0] or [1] |
|  |  | Capsicum annum | Salonaceae | C8.1.25 Green pepper | [0] or [1] |
|  |  | Bacella alba | Basellaceae | C8.1.26 Ceylon spinach | [0] or [1] |
|  |  |  |  | **(C8/set 2) C8.2 Other fruits** |  |
|  |  |  |  | [alert box : Note for interviewer] : หน้า 81  This group includes  [1] This group includes all fruits other than those classified as vitamin A-rich.  [2] Fruits that are sweetened with sugar or canned/packaged in syrup |  |
|  |  |  |  | [alert box : Note for interviewer] :  This group “not” includes  [1] Fruit pies and pastries  [2] processed fruits such as “fruit leathers” are not included and are grouped with “Sentinel sweet foods” (Unhealthy food consumption) |  |
|  |  | *Citrus sinesis* | *Rutaceae* | C8.2.1 Orange | [0] or [1] |
|  |  | *Psidum* | *Myrtaceae* | C8.2.2 Guava | [0] or [1] |
|  |  | *Musa indica* | *Musaceae* | C8.2.3 Banana | [0] or [1] |
|  |  | *Malus domestica* | *Rosaceae* | C8.2.4 Apple | [0] or [1] |
|  |  | *Mangifera indica* | *Anacardiaceae* | C8.2.5 Mango, unripe | [0] or [1] |
|  |  | *Carica papaya* | *Caricaceae* | C8.2.6 Papaya, unripe (green) | [0] or [1] |
|  |  | *Citrullus lanatus* | *Cucurbitaceae* | C8.2.7 Watermelon | [0] or [1] |
|  |  | *Vitis* | *Vitaceae* | C8.2.8 Grapes | [0] or [1] |
|  |  | *Ananas* | *Bomeliaceae* | C8.2.9 Pineapple | [0] or [1] |
|  |  | *Citrus grandis* | *Rutaceae* | C8.2.10 Pomelo | [0] or [1] |
|  |  | *Actinidia deliciosa* | *Actinidiaceae* | C8.2.11 Kiwi | [0] or [1] |
|  |  | *Prunus* | *Rosaceae* | C8.2.12 Strawberry | [0] or [1] |
|  |  | *Nephelium lappaceum* | *Sapindaceae* | C8.2.13 Rambutan | [0] or [1] |
|  |  | *Averrhoa* | *Oxalidaceae* | C8.2.14 Star fruit | [0] or [1] |
|  |  | *Annona squamosa* | *Annonaceae* | C8.2.15 Sweetsop | [0] or [1] |
|  |  | *Tamarindus indica* | *Caesalpinioideae* | C8.2.16 Tamarind | [0] or [1] |
|  |  | *Cucunis melo* | *Cucurbitaceae* | C8.2.17 Honeydew melon | [0] or [1] |
|  |  | *Cocos nucifera* | *Arecaceae* | C8.2.18 Coconut flesh | [0] or [1] |
|  |  | *Durio* | *Malvaceae* | C8.2.19 Durian | [0] or [1] |
|  |  | *Artocarpus heterophyllus* | *Moraceae* | C8.2.20 Jackfruit | [0] or [1] |
|  |  | *Ziziphus jujuba* | *Rhamnaceae* | C8.2.21 Jujube | [0] or [1] |
|  |  | *Manikara zapota* | *Sapotaceae* | C8.2.22 Sapodella | [0] or [1] |
|  |  | *Citrus aurantifolia* | *Rutaceae* | C8.2.23 Lime | [0] or [1] |
|  |  | *Litchi chinensis* | *Sapindaceae* | C8.2.24 Litchi | [0] or [1] |
|  |  | *Morus nigra* | *Moraceae* | C8.2.25 Mulberry | [0] or [1] |
|  |  | *Prunus persica* | *Rosaceae* | C8.2.26 Peach | [0] or [1] |
|  |  | *Pyrus communis* | *Rosaceae* | C8.2.27 Pear | [0] or [1] |
|  |  | *Prunus* | *Rosaceae* | C8.2.28 Plum | [0] or [1] |
|  |  | *Punica granatum* | *Luthraceae* | C8.2.29 Pomegranate | [0] or [1] |
|  |  | *Syzigium malaccense* | *Myrtaceae* | C8.2.30 Pomerac | [0] or [1] |
|  |  | *Saccharum sinense, Saccharum barberi, etc.* | *Poaceae* | C8.2.31 Sugar cane | [0] or [1] |
|  |  | *Prunus domesticus* | *Rosaceae* | C8.2.32 Prune | [0] or [1] |
|  |  | *Cydonia oblongata* | *Rosaceae* | C8.2.33 Quince | [0] or [1] |
|  |  | *Corneus* | *Cornaceae* | C8.2.34 Cherries | [0] or [1] |
|  |  | *Ribes nigrum* | *Grassulariaceae* | C8.2.39 Black current | [0] or [1] |
|  |  | *Citrus limon* | *Rutaceae* | C8.2.40 Lemon | [0] or [1] |
| 3. Minimum meal frequence / MFF |  |  |  | Percentage of children consumed solid, semi-solid or soft foods (but also including milk feeds for non-breastfed children) the minimum number of times or more** | [ ] yes / [ ] no |
| 4. Minimum milk feeding frequency for non-breastfed / MMFF |  |  |  | Percentage of children consumed at least two milk feeds | [ ] yes / [ ] no |
| 5. Minimum acceptable diet / MAD |  |  |  | Percentage of children consumed a minimum acceptable diet |  |
| 6. Egg and/or flesh food consumption / EFF |  |  |  | Percentage of children consumed egg and/or flesh food |  |
| 7. Sweet beverage consumption / SwB | **D** |  |  | [alert box : inform participant]  Now I would like to ask you about sweet beverage that [NAME] had yesterday during the day or at night.  Whether made at home, by informal vendors or packaged in cans, bottles, boxes, sachets, etc. |  |
|  |  |  |  | **D1 Sweet or flavoured type of milk / yogurt / yogurt drinks** |  |
|  |  |  |  | [alert box : Note for interviewer]  Note for interviewer: non-dairy creamers whether liquid or powdered also do not count in this line item |  |
|  |  |  |  | D1.1 Flavoured milks | [0] or [1] |
|  |  |  |  | D1.2 milk chocolate | [0] or [1] |
|  |  |  |  | D1.3 condensed milk | [0] or [1] |
|  |  |  |  | D1.4 smoothies | [0] or [1] |
|  |  |  |  | D 1.5 milkshakes | [0] or [1] |
|  |  |  |  | D 1.6 whipped cream | [0] or [1] |
|  |  |  |  | D1.7 sweetened yogurt | [0] or [1] |
|  |  |  |  | D1.8 sweetened drinking yogurt | [0] or [1] |
|  |  |  |  | **D2 Any type of fruit juice** |  |
|  |  |  |  | D2.1 100% fruit juice | [0] or [1] |
|  |  |  |  | D2.2 fruit-flavoured drink | [0] or [1] |
|  |  |  |  | D2.3 fruit squash | [0] or [1] |
|  |  |  |  | **D3 “Sodas” means sweetened carbonated beverages.** |  |
|  |  |  |  | D3 Soda | [0] or [1] |
|  |  | . |  | **D4 All of these beverages are assumed to have sugar or sweeteners.** |  |
|  |  |  |  | [alert box : inform participant]  Beverages in this group include home-made drinks of any kind to which sweeteners (e.g., sugar, honey, syrup, flavoring powders) have been added. |  |
|  |  |  |  | D4 beverages are assumed to have sugar or sweeteners. | [0] or [1] |
| 8. Unhealthy food consumption / UFC | **E** |  |  | **E Sentinel unhealthy foods** |  |
|  |  |  |  | alert box : inform participant]  **Now I would like to ask you about Sentinel unhealthy foods that [NAME] had yesterday during the day or at night.** |  |
|  |  |  |  | [alert box : Note for interviewer] หน้า 13  Consumption of any amount of food from any of the sentinel categories “counts”, i.e., there is no minimum quantity. |  |
|  |  |  |  | **E1 Sugar confections** |  |
|  |  |  |  | E1.1 Chocolates | [0] or [1] |
|  |  |  |  | E1.2 Caramels | [0] or [1] |
|  |  |  |  | E1.3 Gummies | [0] or [1] |
|  |  |  |  | E1.4 Jelly | [0] or [1] |
|  |  |  |  | E1.5 Candies | [0] or [1] |
|  |  |  |  | E1.6 Toffee | [0] or [1] |
|  |  |  |  | E1.7 Marshmallow | [0] or [1] |
|  |  |  |  | E1.8 Fruit roll-ups | [0] or [1] |
|  |  |  |  | E1.9 Fondant | [0] or [1] |
|  |  |  |  | **E2** **Sweet baked or fried confections** |  |
|  |  |  |  | [alert box : Note for interviewer]  Sweet baked or fried confections are included those made with real fruit or vegetables or nuts, like apple cake or cherry pie. |  |
|  |  |  |  | E2.1 Cakes | [0] or [1] |
|  |  |  |  | E2.2 Cookies | [0] or [1] |
|  |  |  |  | E2.3 Sweet biscuits | [0] or [1] |
|  |  |  |  | E2.4 Doughnuts | [0] or [1] |
|  |  |  |  | E2.5 Pastries e.g., Pies Croissant tart | [0] or [1] |
|  |  |  |  | **E3 Frozen desserts or treats e.g., gelato, sherbet, sorbet, popsicle** |  |
|  |  |  |  | E3 Frozen desserts or treats | [0] or [1] |
|  |  |  |  | **E4 Sentinel fried and salty foods** |  |
|  |  |  |  | E4.1 Chips | [0] or [1] |
|  |  |  |  | E4.2 French fries | [0] or [1] |
|  |  |  |  | E4.3 Crisp snack | [0] or [1] |
|  |  |  |  | E4.4 Fried plantain snacks | [0] or [1] |
|  |  |  |  | E4.5 Instant noodles* | [0] or [1] |
| 9. Zero vegetable or fruit consumption / ZVF |  |  |  | Percentage of children did not consume any vegetables or fruits |  |
| 10. Bottle feeding  /BoF |  |  |  | Did [NAME] drink anything from a bottle with a nipple yesterday during the day or at night?  This indicator is based on consumption of any food or drink from a bottle with a nipple/ teat (including breast milk). |  |
|  |  |  |  | alert box : inform participant]  Some foods have characteristics that increase the risk that they will obstruct the airway, such as being small, smooth, round or cylindrical, flexible, or compressible. These foods include uncooked or whole foods such as pulses, beans, and peas, as well as foods that are simple to bite, such as cucumbers, apples, and carrots. You should modify the size, shape, and texture of this food group before serving it to your child. |  |

*Additional food items

PART 4. food and milk consumption frequency (F, H)

| Indicators / short name | SET | Question | Response |
| --- | --- | --- | --- |
| จากคำถามในแต่ละข้อ เอามาคำนวณตอบ indicator ต่อไปนี้  1. Continued breastfeeding / CBF  2. Minimum dietary diversity / MDD  (2.1 breast milk)  3. Minimum meal frequence / MFF  4. Minimum milk feeding frequency for non-breastfed children/ MMFF |  | Feed frequency |  |
|  |  | [alert box : inform participant]  Now I would like to ask you about the fed milk that [NAME] had yesterday during the day or night |  |
|  |  | F Breastfeeding frequency |  |
|  |  | [alert box : inform participant]  Now I would like to ask you about the fed breast milk that [NAME] had yesterday during the day or night (breastfeeding, including by a wet nurse, and feeding of expressed breast milk). |  |
|  | F1 | F1 Was [NAME] fed breast milk yesterday during the day or at night?  1. Yes  0. No | > If (F1) Yes [1]  : Ask F2.1-F2.6 |
|  |  |  | [ 0 ]  > If (H1) No[ ]  : Skip to G |
|  | F 2 | F2 Feeding frequency with breast milk |  |
|  |  | F2.1 "How many times during the night does [NAME] take breast milk by wet nursing?" | [A] Number  I___I  [B] |
|  |  | F2.2 "How many times during the night does [NAME] take breast milk by bottle with nipple feeding?" | [A] Number  I___I  [B] |
|  |  | F2.3 "How many times during the night does [NAME] take breast milk by other method (e.g., sippy cup, cup)?" | [A] Number  I___I  [B] |
|  |  | F2.4 "How many times during the daytime does [NAME] take breast milk by wet nursing?" | [A] Number  I___I  [B] |
|  |  | F2.5 "How many times during the daytime does [NAME] take breast milk by bottle with nipple feeding?" | [A] Number  I___I  [B] |
|  |  | F2.6 "How many times during the daytime does [NAME] take breast milk by other method (e.g., sippy cup, cup)?" | [A] Number  I___I  [B] |
|  | G | G Milk feeds frequency |  |
|  |  | [alert box : inform participant]  Now I would like to ask you about the fed milk other than breastmilk that [NAME] had yesterday during the day or night |  |
|  |  | [alert box : Note for interviewer]  Milk feeds included  1. Infant formula, such as *[insert local*  *names of common formula]*?  Any type of commercially-produced infant formula including soy formula, predigested/hydrolysed formula and “follow-up” formula (growing-up milk, toddler milk), but not homemade mixtures.  2. Milk from animals such as fresh, tinned  or powdered milk?  Any type of milk from any mammal (e.g., cow, goat, sheep, buffalo, camel) which is given to the child as a liquid (drink). This includes liquid milk whether raw or pasteurized, reconstituted powdered milk, or evapourated milk (tinned).  Milk feeds “not” included:  1. Flavored milks, milkshakes or other animal milk-based shakes and smoothies are also included.  2. Dairy cream (e.g., half and half, whipping cream)  3. Sweetened condensed milk  4. Does not include yogurt that is eaten with a spoon rather than drunk |  |
|  | G1 | G1 Did [NAME] drink formula milk other than breastmilk?  1. Yes  0. No | [ 1 ]  > If (H1) Yes [1]  : Ask G2.1-G2.4 |
|  |  |  | [ 0 ]  > If (H1) No[0] : Skip to H |
|  | G2 | G2 Feeding frequency with milk (other than breastmilk). | ~~[A] Number~~  ~~I___I~~  ~~[B]~~ |
|  |  | G2.1 How many times did [NAME] drink formula (milk other than breastmilk during the night by bottle with nipple feeding? | [A] Number  I___I  [B] |
|  |  | G2.2 How many times did [NAME] drink formula (milk other than breastmilk during the night by other method (e.g., sippy cup, cup)? | [A] Number  I___I  [B] |
|  |  | G2.3 How many times did [NAME] drink formula (milk other than breastmilk during the daytime by bottle with nipple feeding? | [A] Number  I___I  [B] |
|  |  | G2.4 How many times did [NAME] drink formula (milk other than breastmilk during the daytime by other method (e.g., sippy cup, cup)? | [A] Number  I___I  [B] |
|  | H | H meal and snack frequency |  |
|  | H | [alert box : inform participant]  Now I would like to ask you about feeding episodes (meals or snacks) that contained non-liquid food. that [NAME] had yesterday during the day or night this question intends to capture information on the frequency of feeding solid, semi-solid or soft foods, i.e., individual feeding episodes (meals or snacks) that contained non-liquid food. |  |
|  |  | [alert box : inform participant]  Not to be considered a feeding episode  1. Very trivial snacks should not be counted as meals or snacks, e.g., a bite of a banana or  2. Contained only liquid food. |  |
|  |  | H1 How many meals that children consumed solid, semi-solid or soft foods yesterday during the night? | [A] Number  I___I  [B] |
|  |  | H2 How many meals that children consumed solid, semi-solid or soft foods yesterday during the day? | [A] Number  I___I  [B] |

PART 5. Barriers to complementary feeding are imposed by the maternal or caregiver perspective (I).

| **SET** | **Question** | **Response** |
| --- | --- | --- |
|  | **F Barriers** |  |
| I | [alert box : inform participant]  After viewing the list of foods that infants and young children should receive as complementary foods, what is stopping you from giving them those foods?  Please select "rate 1-5 "  [1] never  [2] sometimes  [3] often  [4] usually  [5] always |  |
| I1 | F1 The child prefers milk to supplemental foods.  [1] never  [2] sometimes  [3] often  [4] usually  [5] always | [1] [2] [3] [4] [5] |
| I2 | F2 The child always refused to eat a new kind of supplemental food.  [1] never  [2] sometimes  [3] often  [4] usually  [5] always | [1] [2] [3] [4] [5] |
| I3 | F3 not confident regarding food types  [1] never  [2] sometimes  [3] often  [4] usually  [5] always | [1] [2] [3] [4] [5] |
| I4 | F4 not confident regarding food amounts  [1] never  [2] sometimes  [3] often  [4] usually  [5] always | [1] [2] [3] [4] [5] |
| I5 | F5 not confident regarding texture of food  [1] never  [2] sometimes  [3] often  [4] usually  [5] always | [1] [2] [3] [4] [5] |
| I6 | F6 the price of complementary foods  [1] never  [2] sometimes  [3] often  [4] usually  [5] always | [1] [2] [3] [4] [5] |
| I7 | F7 Lack of control over the choice of complementary foods because of cultural elders' influence/culture  [1] never  [2] sometimes  [3] often  [4] usually  [5] always | [1] [2] [3] [4] [5] |

**Web-Based Application – Page Descriptions**

| **Homepage**  Contains five sections, each presenting a set of questions. |
| --- |
| 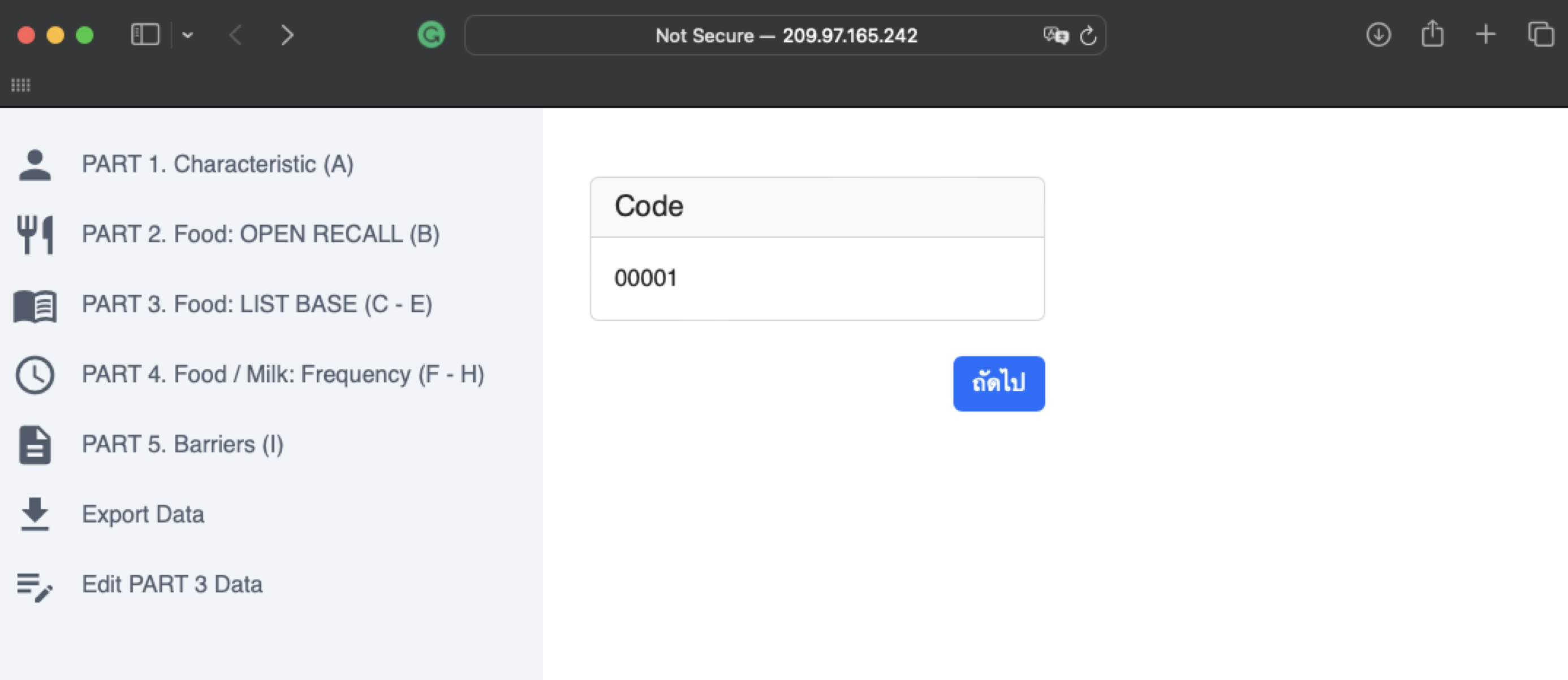 |
| **Pop-Up Boxes**:  **Green Pop-Up Box**: Indicates content that the interviewer must read aloud to the participant.  **Orange Pop-Up Box**: Serves as a reminder or prompt for the interviewer. |
| 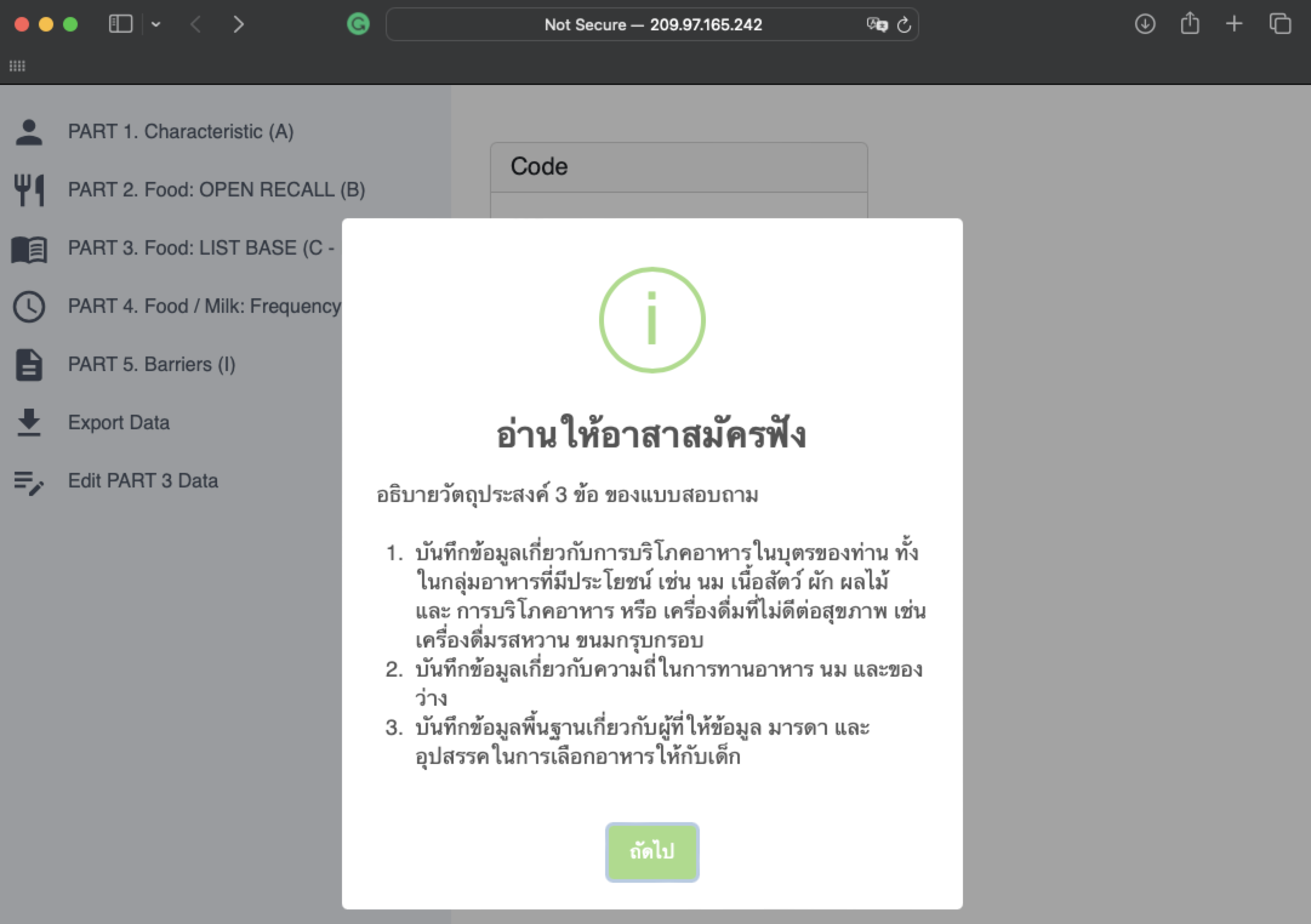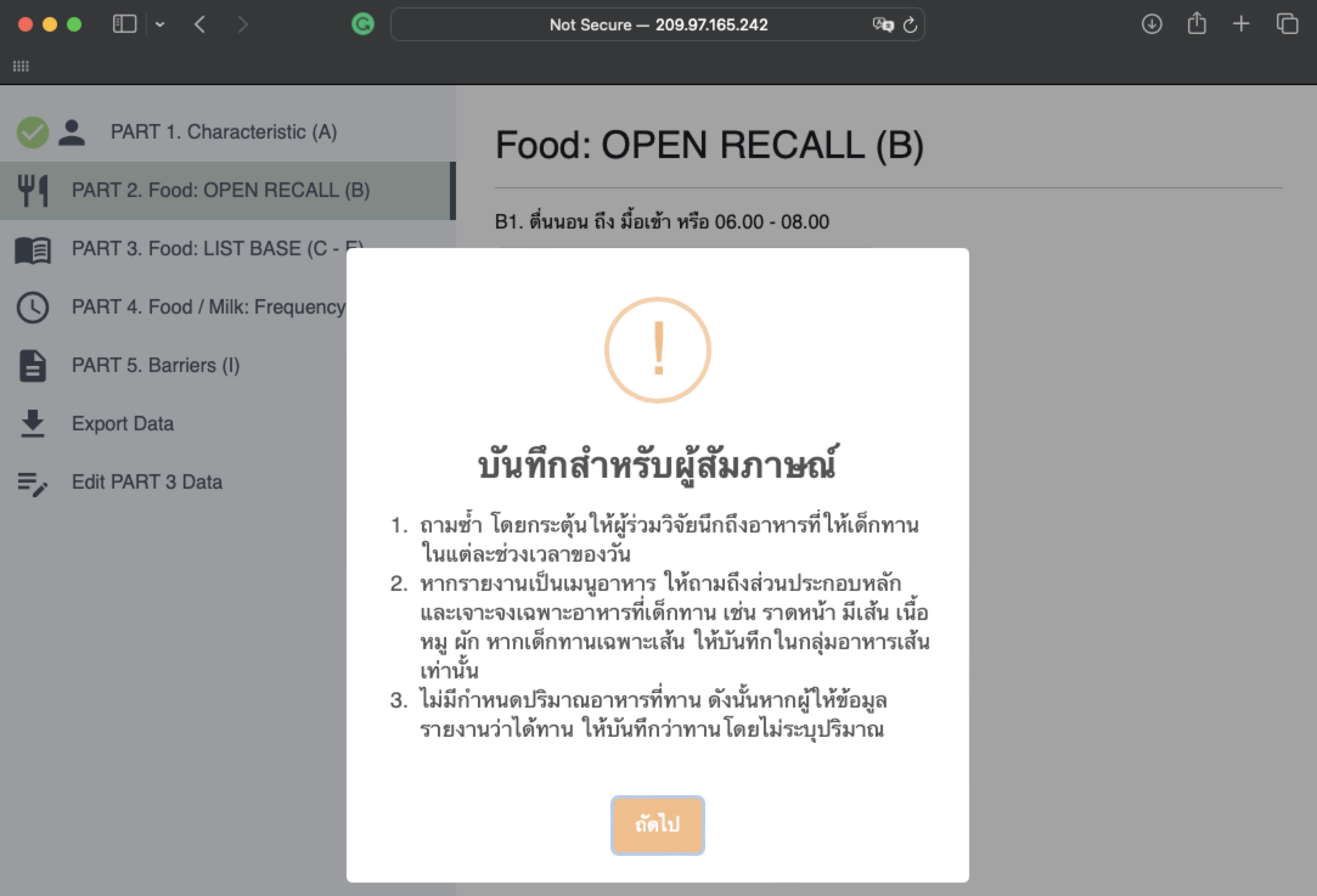 |
| **Part 1 Characteristics and Information Sources**:  Collects details about the participant’s characteristics and the sources of complementary feeding information. |
| 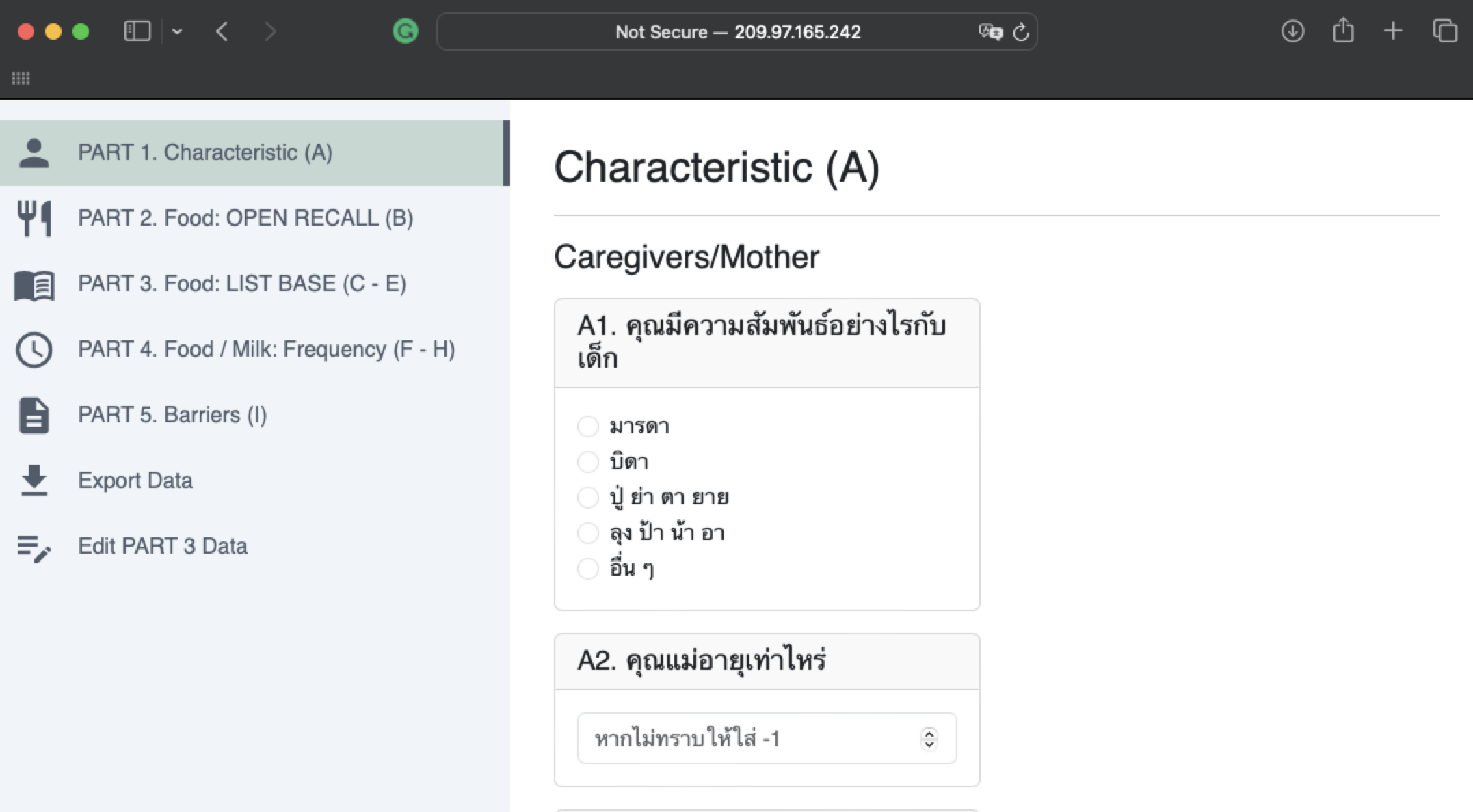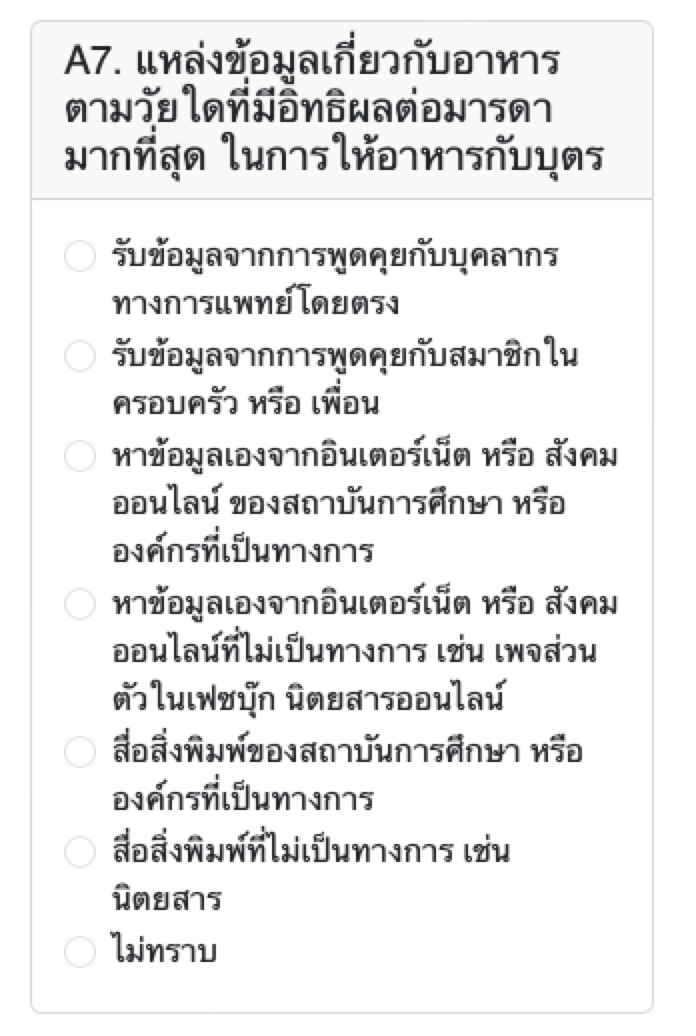 |
| **Part 2 Open Recall**  The interviewer asks about the foods consumed by the child throughout different periods of the day.  When entering a portion of a food name into the dropdown menu, the system suggests previously recorded food items to prevent duplicate entries in subsequent steps. |
|  |
| 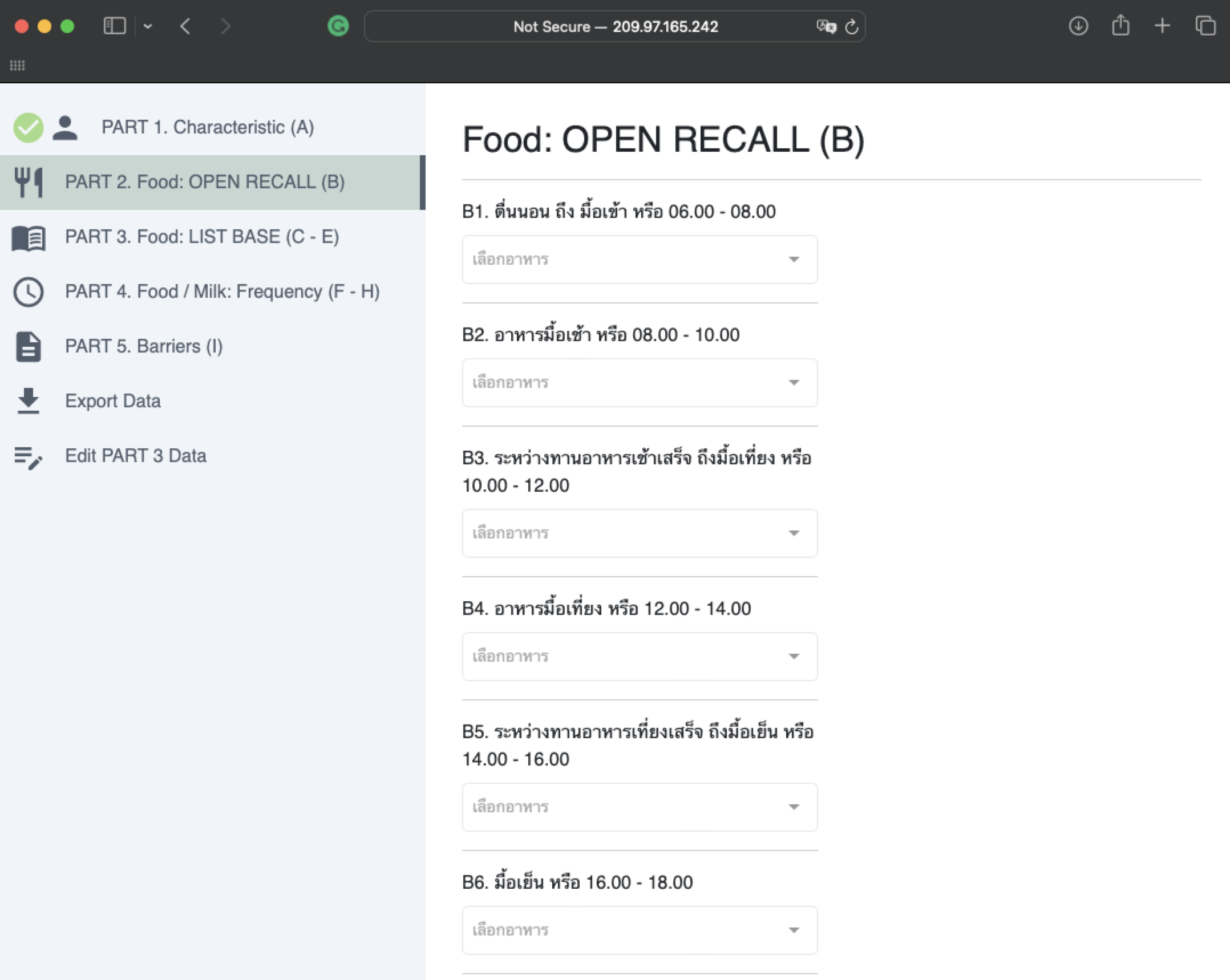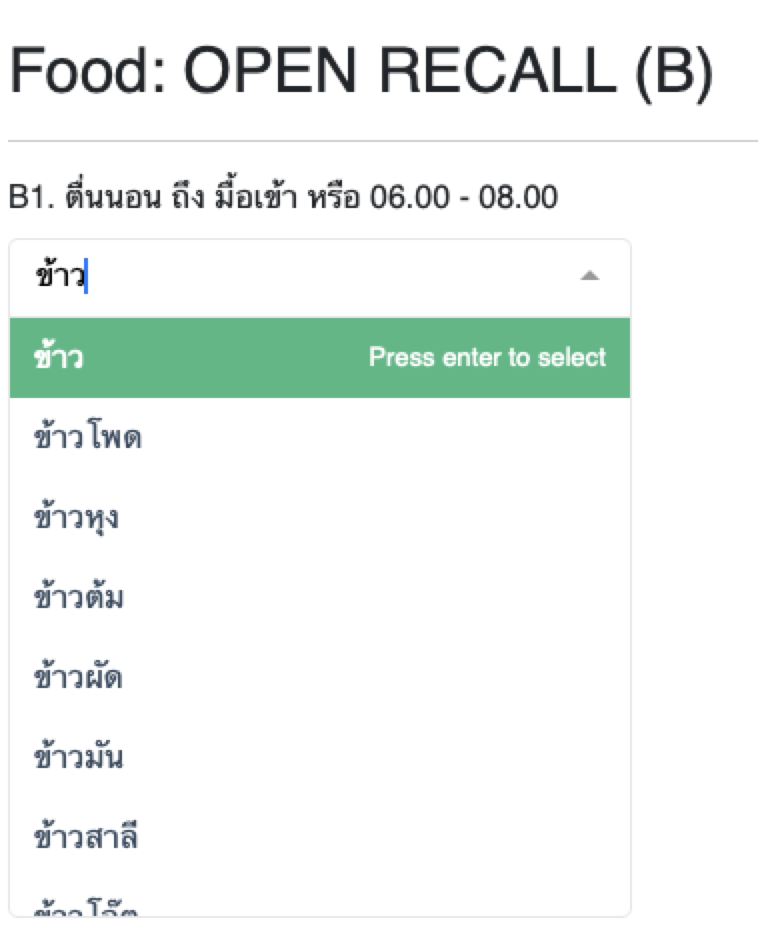 |
| **Part 3 Food list base**  The interviewer reads aloud food items that have not yet been reported.  If a food is not on the predefined list, it is recorded under the "Other" section.  A nutritionist later classifies and manually edits the food group assignments. |
| 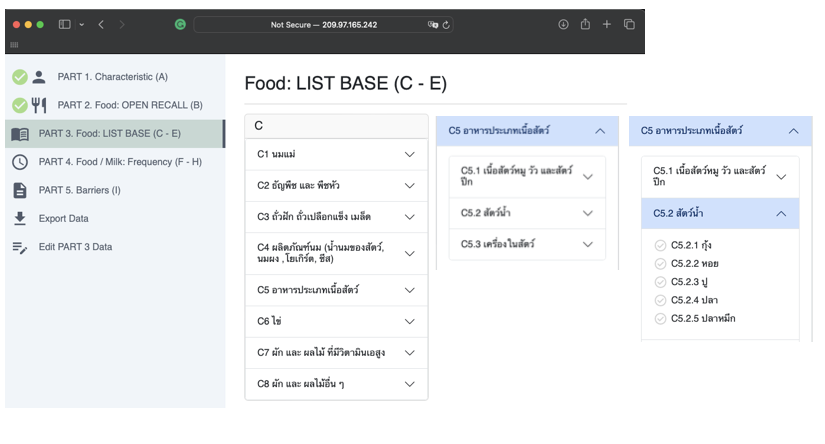 |
| **Part 4 Feeding Frequency 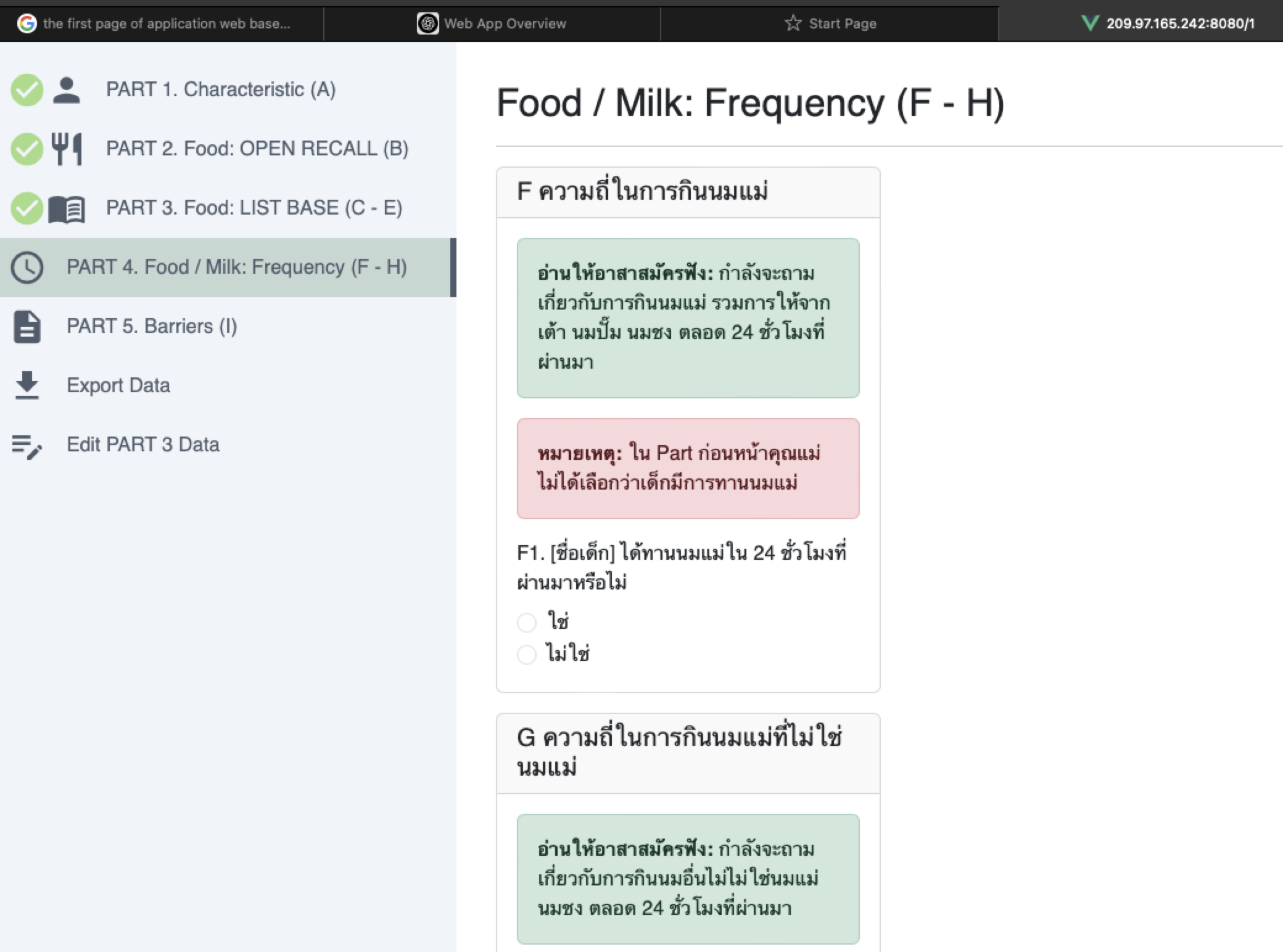**  **Part 5 Barrier**  The interviewer reads statements, and the participant provides a rating based on their experience. |
| 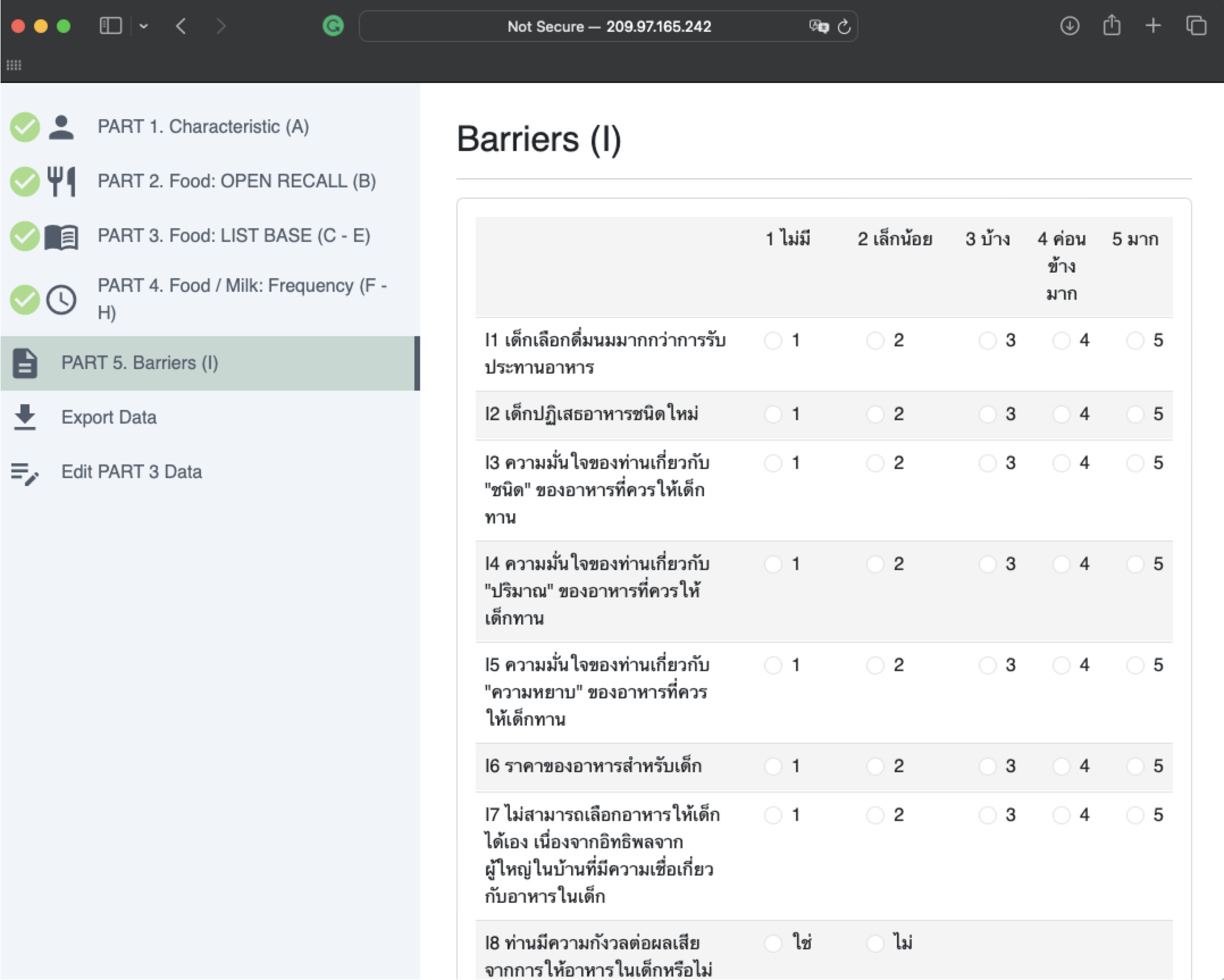 |

**Operational Definition: Complementary Feeding Indicators**Definitions were adapted from the World Health Organization and the United Nations Children’s Fund (UNICEF). *Indicators for Assessing Infant and Young Child Feeding Practices: Definitions and Measurement Methods*. Geneva: WHO and UNICEF; 2021.

Continued breastfeeding CBF : Percentage of children who were fed breast milk during the previous day (This deviates from the original WHO indicator, which applies only to children aged 12 to 24 months, but in this study, the indicator was applied to children aged 6 to 24 months.)

Minimum dietary diversity MDD : Percentage of children who consumed foods and beverages from at least five out of eight defined food groups during the previous day

Minimum meal frequency (MMF): Percentage of children who consumed solid, semi-solid or soft foods (but also including milk feeds for non-breastfed children) the minimum number of times or more during the previous day

Minimum milk feedingfrequency for non-breastfed children (MMFF): Percentage of non-breastfed children who consumed at least two milk feeds during the previous day

Minimum acceptable diet (MAD): Percentage of children who consumed a minimum acceptable diet during the previous day

Egg and/or flesh food consumption (EFF): Percentage of children who consumed egg and/or flesh food during the previous day

Sweet beverage consumption (SwB): Percentage of children who consumed a sweet beverage during the previous day

Unhealthy food consumption (UFC): Percentage of children who consumed selected sentinel unhealthy foods during the previous day

Zero vegetable or fruit (ZVF): Percentage of children who did not consume any vegetables or fruits during the previous day

Bottle feeding (BoF): Percentage of children who were fed from a bottle with a nipple during the previous day
